# An Integrated Approach Including CRISPR/Cas9-Mediated Nanopore Sequencing, Mate Pair Sequencing, and Cytogenomic Methods to Characterize Complex Structural Rearrangements in Acute Myeloid Leukemia

**DOI:** 10.3390/biomedicines12030598

**Published:** 2024-03-07

**Authors:** Michael Phan, Maria A. Gomes, Victoria Stinnett, Laura Morsberger, Nicole L. Hoppman, Kathryn E. Pearce, Kirstin Smith, Brian Phan, Liqun Jiang, Ying S. Zou

**Affiliations:** 1 Krieger School of Arts and Sciences, Johns Hopkins University, Baltimore, MD 21205, USA; mphan4@jhu.edu; 2 Mount Hebron High School, Ellicott City, MD 21042, USA; anitautsw@gmail.com; 3 Department of Pathology, School of Medicine, Johns Hopkins University, Baltimore, MD 21205, USA; vlloyd3@jhmi.edu (V.S.); lmorsber@jhmi.edu (L.M.); ksmith83@jhmi.edu (K.S.); liqunjiang@gmail.com (L.J.); 4 Department of Laboratory Medicine and Pathology, Mayo Clinic, Rochester, MN 55902, USA; hoppman.nicole@mayo.edu (N.L.H.); pearce.kathryn@mayo.edu (K.E.P.); 5 Department of Biology, The College of William and Mary, Williamsburg, VA 23185, USA; bphan3@jh.edu

**Keywords:** CRISPR/Cas9, nanopore sequencing, mate pair sequencing, chromoanagenesis, complex structural abnormalities, acute myeloid leukemia

## Abstract

Complex structural chromosome abnormalities such as chromoanagenesis have been reported in acute myeloid leukemia (AML). They are usually not well characterized by conventional genetic methods, and the characterization of chromoanagenesis structural abnormalities from short-read sequencing still presents challenges. Here, we characterized complex structural abnormalities involving chromosomes 2, 3, and 7 in an AML patient using an integrated approach including CRISPR/Cas9-mediated nanopore sequencing, mate pair sequencing (MPseq), and SNP microarray analysis along with cytogenetic methods. SNP microarray analysis revealed chromoanagenesis involving chromosomes 3 and 7, and a pseudotricentric chromosome 7 was revealed by cytogenetic methods. MPseq revealed 138 structural variants (SVs) as putative junctions of complex rearrangements involving chromosomes 2, 3, and 7, which led to 16 novel gene fusions and 33 truncated genes. Thirty CRISPR RNA (crRNA) sequences were designed to map 29 SVs, of which 27 (93.1%) were on-target based on CRISPR/Cas9 crRNA nanopore sequencing. In addition to simple SVs, complex SVs involving over two breakpoints were also revealed. Twenty-one SVs (77.8% of the on-target SVs) were also revealed by MPseq with shared SV breakpoints. Approximately three-quarters of breakpoints were located within genes, especially intronic regions, and one-quarter of breakpoints were intergenic. Alu and LINE repeat elements were frequent among breakpoints. Amplification of the chromosome 7 centromere was also detected by nanopore sequencing. Given the high amplification of the chromosome 7 centromere, extra chromosome 7 centromere sequences (tricentric), and more gains than losses of genomic material, chromoanasynthesis and chromothripsis may be responsible for forming this highly complex structural abnormality. We showed this combination approach’s value in characterizing complex structural abnormalities for clinical and research applications. Characterization of these complex structural chromosome abnormalities not only will help understand the molecular mechanisms responsible for the process of chromoanagenesis, but also may identify specific molecular targets and their impact on therapy and overall survival.

## 1. Introduction

Complex structural chromosome abnormalities have been reported in myeloid malignancies such as acute myeloid leukemia (AML) and myelodysplastic syndrome (MDS). Complex and massive chromosomal and genomic rearrangements can be generated by a chromoanagenesis event, which is characterized by the simultaneous occurrence of multiple structural alterations through a single catastrophic cellular event at one or more loci [1]. Chromoanagenesis comprises three distinct genomic rearrangements: chromothripsis, chromoanasynthesis, and chromoplexy, with each genomic rearrangement having its mechanism of formation and etiology [2]. In chromothripsis, the driving force behind the phenomenon is through multiple double-strand breaks (DSBs) with deletions in a single catastrophic event that subsequently reassembles chromosomal fragments at random to develop complex derivative chromosomes [3,4,5]. These chromosomes may include additional gain or loss of genetic material from multiple or single chromosomes that lead to alterations in the genomic structure. Analysis of the breakpoint sequences indicates that the rejoining of DNA fragments is likely through non-homologous end joining (NHEJ) or alternative end joining (alt-EJ) [6,7,8]. Random rearrangements in these events often disrupt tumor suppressors and amplify oncogenes present [1]. In chromoanasynthesis, chaotic and complex rearrangements lead to an increase in the copy number (CN) of chromosomes due to interference of stability and stress at the replication forks during DNA replication, resulting in replication errors [9,10,11,12]. Commonly observed replication errors involve serial fork stalling and template switching (FoSTeS) or microhomology-mediated break-induced relocation (MMBIR) mechanisms that lead to region-focused duplications or triplications at the breakpoint junctions [10,13]. In chromoplexy, genomic rearrangement is driven by the multiple inter- and intrachromosomal translocations and deletions at fusion junctions [14]. Unlike chromoanasynthesis or chromothripsis, this phenomenon shows little to no copy number alterations. Evidence to date suggests chromothripsis to be the most probable mechanism underlying most genomic rearrangements in cancers [1].

Chromoanagenesis has been seen across many different forms of cancer with a prevalence of 2–3% [3,15,16,17,18,19,20,21]. The frequency is elevated, however, when specific tumors are considered. The frequency of the event has been seen to reach 25% in bone cancers [3] or 18% during the late stages of neuroblastomas [21]. In rare instances, chromoanagenesis has been responsible for creating one or many cancer-inducing lesions that provide cellular growth through three key routes. The first is the formation of circular DNA fragments that lack centromeres or telomeres but harbor oncogenes (double minute chromosomes) through chromothripsis and NHEJ, facilitating the amplification of oncogenes and cell proliferation [22]. The second is the loss or disruption of tumor suppressor genes through chromothripsis and NHEJ rearrangement. The third is the fusion of oncogenes by joining coding portions of two oncogenes in the same orientation [1]. Previous studies have also indicated a strong relationship between chromoanagenesis and *TP53* mutations in AML. Between chromoanagenesis and *TP53* mutations, whole-genome sequencing (WGS) and microarray analysis revealed germline and somatic inactivation of the *TP53* suppressor gene [16]. In patients with newly diagnosed multiple myeloma, those with genomic rearrangement through chromothripsis revealed an aggressive disease course and poor prognosis, indicating chromothripsis may defines a rare entity of high-risk patients [20].

Chromosomal abnormalities are important for tumor formation and development. These chromosomal abnormalities are responsible for changes in the expression of or function of RNA and proteins, promoting tumor proliferation that affects the immune system, and amplification or deletion that reshapes the genome and influences tumor progression. Chromosomal abnormalities are a shared characteristic among cancers and are categorized as numerical or structural abnormalities [23]. Numerical abnormalities mainly consist of aneuploidy (loss or gain of a region or chromatid) or chromosome instability (CIN) caused by segregation errors during mitosis [24]. Aneuploidy can occur as segmental parts of the genome or as a whole. CIN is one of the leading causes of tumor evolution, leading to a poor survival rate in various malignancies. CIN resulting in tri- or tetraploidy has been known to promote oncogenesis and, in most cases, leads to copy number alterations resulting in aneuploidy with tetraploidy as a common temporary state of aneuploidy. Approximately 90% of human solid tumors and approximately 75% of hematopoietic cancers experienced aneuploidy [25].

Structural abnormalities consist of DNA damage in addition to the gain or loss of genomic material, forming derivative chromosomes. Commonly observed abnormalities range from deletions of chromosomal arms and amplification of genomic regions to alterations of multiple chromosomes [26,27]. The most frequent changes noted have been from deletions, followed by amplification and then unbalanced translocations [28]. Amplification or deletion along the genome has been observed in 88% of cancer samples [29]. Other common structural abnormalities include the gain of genetic material on the q arm of chromosome 8 (33% of cancer samples) and the deletion of genetic material on the p arm of chromosomes 8 and 17 (33% and 35% of cancer samples, respectively). Evidence to date revealed chromosome 2 as the least altered, with aberrations of the p and q arms observed in 18% and 16% of cancer samples, respectively [23]. Previous data have also shown structural abnormalities associated with immune signatures, with 3p, 8p, 13q, and 17p deletions having a positive correlation and 4q, 5q, and 14q deletions having a negative correlation [23]. Structural abnormalities vary in different types of malignancies, with some aberrations seen more frequently and consistently in specific cancers, e.g., the relationship between AML and abnormalities of chromosomes 5 and 7.

For hematological malignancies, recurrent genetic abnormalities are important in classifying AML, e.g., the French–American–British (FAB) system. Common chromosomal abnormalities in AML include t(8;21) translocation, t(15;17) translocation, inversion of chromosome 16, monosomy of chromosome 5/5q deletion, monosomy of chromosome 7/7q deletion, or trisomy of chromosome 8 [30].

Common methods used in identifying chromothripsis are fluorescence in situ hybridization (FISH) analysis, SNP microarray analysis, and recently, next-generation sequencing (NGS) assays used to detect multiple CN states at each clustering breakpoint location [31,32,33]. Chromothripsis in AML has been detected on chromosomes 3, 5, 6, 7, 8, 10, 11, 12, 15, 17, and 20 with structural changes that include deletions of 4q28–-32, 7q31.1–36.3, 12p11.21–13.3, 16q22–24.3, 17p13–13.1, and 5q31.1–33.1 [31,32]. Given the formation of ring chromosomes has been frequently associated with mutations in *TP53* [34], studies have also noted a strong relationship between chromothripsis and these ring chromosomes carrying a mutation in *TP53* [31]. Reported genes that are often involved in a chromothripsis event have been amplified by *MYC* and *KMT2A*. Approximately 6.6% of de novo AML cases have been reported with chromothripsis [31].

Despite improvements in NGS-based genomics technology, the detection of complex structural chromosome abnormalities from short-read sequencing still poses challenges. The challenge of short-read sequencing is within the read length, as a short read length does not allow full representation of the human genome. Short read lengths cause the inability to read certain regions in the human genome, such as centromere regions, telomeres, and acrocentric genomic regions with tandem repeats [35]. As a result, a higher mutation rate cannot be read, leaving an incomplete understanding of the human genome [36,37,38,39]. Furthermore, short-read sequencing limits our understanding of complex relationships that occur in chromoanagenesis.

Long-read sequencing approaches, such as Oxford nanopore technology (ONT, Oxford, UK), are promising for characterizing chromosomal abnormalities. Although long-read sequencing poses new challenges, many of the shortcomings with short-read sequencing are resolved. Long-read sequencing allows the proper identification of simple structural abnormalities along with a better understanding of long-range structural abnormalities that occur in chromoanagenesis. Long-read sequencing can sequence stretches of DNA of up to hundreds of kilobases in length [40,41]. Furthermore, clustered regularly interspaced short palindromic repeats (CRISPR)/Cas9-mediated nanopore sequencing is used for amplifying targeted sequences containing our desired genomic region of interest. CRISPR/Cas9 is a novel gene-editing technique that can efficiently induce targeted genetic modifications. Compared to polymerase chain reaction (PCR), it is more cost-efficient and allows a higher mapping quality [42]. CRISPR/Cas9 with nanopore sequencing provides greater sensitivity, allowing for real-time sequencing of the DNA, compared to nanopore sequencing itself, which has high error rates [42]. This detection method allows structural variants (SVs) to exist within our sequence of interest. For the CRISPR/Cas9 ribonucleoprotein complexes, the sequence of the guide ribonucleic acid (guideRNA) is custom-designed. The guideRNA serves to recognize specific sequences of the DNA, where the ends of the cut site would be ligated to a sequencing adaptor, which then allows the region of interest to be sequenced [42].

Mate pair sequencing (MPseq) allows better detection of chromosomal abnormalities. After the genomic DNA is first fragmented, biotin is added to these ends, which then allows the fragmented DNA to circularize. The circularization method during this process allows for the detection of its SVs [43,44]. MPseq’s resulting coverage data can also be used to identify the copy number alterations, where we can identify the gain or loss of a copy number variant (CNV) within the genome. Through this technique, we can better identify the relationship among genetic materials even from different chromosomes.

In this study, we used an integrated approach including CRISPR/Cas9-mediated nanopore sequencing, MPseq, and SNP microarray analysis, along with conventional cytogenetic methods (chromosome analysis and FISH), to characterize complex structural chromosome abnormalities (chromoanagenesis involving chromosomes 2, 3, and 7) in AML. We have demonstrated the value of this combination approach in characterizing complex structural abnormalities for clinical and research applications.

## 2. Materials and Methods

### 2.1. Patient Data and Diagnosis of Acute Myeloid Leukemia/Myelodysplastic Syndrome

A male patient presented with shortness of breath and was found to have pancytopenia with circulating blasts on a smear. Acute myeloid leukemia/myelodysplastic syndrome was diagnosed by bone marrow morphology, immunostaining, and flow cytometry. Flow cytometry of the peripheral blood showed 5% phenotypically abnormal cells and an unusual myeloid blast population expressing CD13, CD24, and CD117 with dim partial CD33 and aberrant CD7. A bone marrow biopsy from his right iliac crest showed 20% atypical cells with unusual myeloid phenotype, expressing CD34, bright CD117, variable HLADR, CD38, CD13, and dim partial CD33, along with partial aberrant CD7.

The surgical pathology results from the bone marrow biopsy classified him as AML. The marrow cellularity was 80–90% and showed an increased population of immature cells. There was residual hematopoiesis with prominent developing erythroid forms with left-shifted granulopoiesis. The ratio of myeloid to erythroid precursors was about 1–2:1. Megakaryocytes were decreased in number with some small, hypolobate forms identified. The aspirate contained sheets of blasts with scant to moderate cytoplasm and distinct nucleoli. There were a few immature myeloid elements with a maturation arrest, and there was erythropoiesis showing dysplastic maturation of nuclear budding and irregular nuclear membranes. An iron stain performed on the clot section showed rare ring sideroblasts, accounting for <5% of cells. CD61 stains identified megakaryocytes and highlighted several micromegakaryocytes not clearly seen on routine stains. CD34 stained about 20% of the blasts, although these were more numerous in some areas. Peripheral blood showed circulating blasts and occasional nucleated erythroid precursors.

All procedures followed were in accordance with the ethical standards of the Institutional Committee on Human Experimentation and with the Helsinki Declaration of 1975. The study was approved by the Local Ethics Committee from the Johns Hopkins Hospital (Baltimore, MD), USA.

### 2.2. Cytogenetics Data: Conventional Chromosome Analysis, FISH, and SNP Microarray

Conventional G-banded chromosome studies were performed using standard techniques. At least 20 metaphase cells were analyzed from unstimulated bone marrow aspirate. The abnormal karyotypes were described using the International System for Human Cytogenetic Nomenclature (2020).

FISH was performed on interphase nuclei from cultured bone marrow cells using disease-specific probes, according to the manufacturer’s instructions (Abbott Molecular Inc., Des Plaines, IL, USA). The specimen was considered abnormal if the results exceeded the laboratory-established cutoff for each probe set.

Whole-genome single-nucleotide polymorphism (SNP) microarray analysis was performed with DNA extracted from bone marrow specimens by conventional methods (Qiacube). The DNA concentration was assessed using a Qubit fluorometer (Thermo Fisher Scientific, Waltham, MA, USA). The high-resolution microarray platform utilized was the Illumina Infinium CytoSNP-850 K v1.2 BeadChip containing > 850,000 markers (mean spacing, 3.5 kb; Illumina, Inc., San Diego, CA, USA). BeadChips were processed per manufacturer’s guidelines and imaged with the Illumina iScan system. Data were analyzed with the CNV Partition 2.4.4.0 algorithm in GenomeStudio version 2010.3 (Illumina) and KaryoStudio version 1.4.3.0 (Illumina). B allele frequency and logR signal intensities were used to examine and identify potential pathogenic regions of genomic imbalance. All analyses were performed using human reference genome assembly hg19 (GRCh37).

### 2.3. Mate Pair Sequencing

DNA extraction and mate pair library preparation methods were performed as previously described [45,46]. MPseq data were mapped to the reference genome GRCh38 using BIMA V3 [47], and SVAtools [46] was used to reveal SVs. Detection of SVs by SVAtools combines three algorithmic approaches: read-pair, split-read, and read depth/count. Clustering of the discordant and split-read fragments was performed by SVAtools to reveal SVs. Only a cluster with more than three fragments, passing the mask/filter criteria, and being called by SVAtools is considered a putative junction.

### 2.4. CRISPR/Cas9-Mediated Nanopore Sequencing

The crRNAs for these SVs were designed using Integrated DNA Technologies (IDT)’s design tool and selected for the highest predicted on-target performance with minimal off-target activity (IDT, Inc., Coralville, IA, USA). GuideRNA was assembled as a duplex from synthetic CRISPR RNAs (crRNAs) (Custom designed, IDT, Inc., Coralville, IA, USA) and tracrRNAs (IDT#1072532). The guideRNA duplex was designed to introduce cuts and to target flanking areas of the region of interest. CRISPR/Cas9-mediated nanopore sequencing and data analysis have been described previously [42]. Briefly, the guideRNA sequence recognizes DNA sequences around the region of interest, where the CAS-9 protein’s endonuclease activity then cuts the 3′ end of the recognized sequence. The now free region of interest has its ends ligated to a sequencing adaptor, which then allows the region of interest to be sequenced. Using this method, thirty crRNAs were designed to detect twenty-nine chromosomal abnormalities in chromosomes 3 and 7 (Supplemental Table S1). crRNA #28 and #29 were targeted for the same genomic region. crRNAs were designed using Custom Alt-R CRISPR-Cas9 guide RNA (https://www.idtdna.com/site/order/designtool/index/CRISPR_CUSTOM, 15 February 2020) and Chopchop (https://chopchop.cbu. uib.no/, 15 February 2020) with CRISPR-Cas9. All analyses were performed using human reference genome assembly GRCh37/hg19, and SVs were reviewed independently by multiple genetic analysts via the Integrative Genomics Viewer (IGV, Broad Institute, Cambridge, MA, USA). Only clusters with more than ten reads at each potential SV breakpoint, with each read having bidirectionality, were considered a putative SV junction. SVs found within 20 kilobases (kbs) of a crRNA sequence were defined as on-target SVs.

### 2.5. Data Comparison among MPseq, Nanopore Sequencing, and SNP Microarray

A comparison of CNV calls from nanopore sequencing and SNP microarray analysis was performed using VIA software version 7.0 (Bionano company, San Diego, CA, USA). SV data by MPseq were converted to the reference genome hg19 (GRCh37) before being compared with SVs detected by nanopore sequencing. All SVs involving chromosomes 2, 3, and 7 by nanopore sequencing were manually reviewed using IGV.

### 2.6. Gene Mutation Panel by Next-Generation Sequencing (NGS)

DNA was extracted by conventional methods per manufacturer’s instructions (QI-Acube; Qiagen, Hilden, Germany). The DNA concentration was assessed using a Qubit fluorometer (Thermo Fisher Scientific, Waltham, MA, USA). NGS was performed on extracted genomic DNA, as outlined previously [48,49]. Briefly, library preparation was performed using Kapa Roche (Wilmington, MA, USA) reagents, hybrid capture was performed using IDT probes (Coralville, IA, USA), libraries were sequenced using an Illumina NovaSeq (paired-end technology; Illumina, San Diego, CA, USA), and sequences were aligned to GRCh37/hg19. The targeted NGS assay used 40,670 IDT probes to cover a panel of 642 pan-cancer genes [48]. The mean read depth was 765× (range 341–1289), and 99.99% of target regions were captured at a level higher than 150×. Sequencing reads were visualized using IGV. As previously described [34], oncogenic somatic variants were considered candidate somatic mutations if (1) variants were present with a minimum variant allele frequency of ≥1%, in at least two alternate reads in both directions, and had an alternate allele base with mean Qscore of ≥11; (2) variants are described in COSMIC and/or ClinVar as being known cancer-associated mutations or mutational hotspots; and (3) variants were classified as deleterious and/or probably damaging by PolyPhen-2 [50] and/or SIFT [51] servers.

## 3. Results

### 3.1. Cytogenetic Results

**Figure 1:**
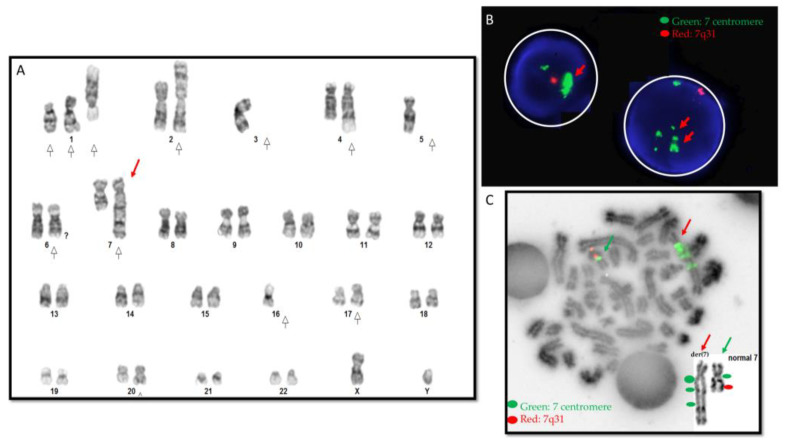
Cytogenetic data. (**A**) Karyogram. Red arrows point to an abnormal derivative chromosome 7, and white arrows point to other numerical and structural abnormalities. (**B**) Interphase FISH revealed amplification of chromosome 7 centromeres (in green color, pointed by red arrows) and deletion of 7q31 (in red color). (**C**) Metaphase FISH. The derivative chromosome 7 (red arrow) shows multiple signals and amplification of the green centromere signal. The green arrow points to a normal chromosome 7. The right-side inserted box shows the make-up of the derivative chromosome 7 by conventional chromosome analysis and FISH data. The derivative chromosome 7 was pseudotricentric and showed centromere amplification.

Conventional chromosome analysis revealed an abnormal karyotype with a complex derivative chromosome 7: 44,XY,add(1)(p12),t(1;4)(p13;q31.1),add(2)(p13),−3,−5,add(6) (q25),der(7)?hsr(7)(q11.2)?hsr(7)(q?22)t(3;7)(p12;q?31),−16,der(16)t(16;17)(q12.1;q11.2),−17, add(17)(p13),del(17)(p13),add(20)(q11.2),der(?)t(?;1)(?;p22)[cp18]/46,XY [2] (Figure 1A). FISH revealed amplification of the chromosome 7 centromere resulting in the derivative chromosome 7 (Figure 1B). FISH also detected monosomy 5 and deletions of 16q22 and 20q12–13.12. FISH results for t(15;17) *PML::RARA*; t(9;22) *BCR::ABL1*, and t(8;21) *RUNX1::RUNX1T1* were normal. Metaphase FISH revealed amplification of the chromosome 7 centromere on the derivative chromosome 7 (Figure 1C).

**Figure 2:**
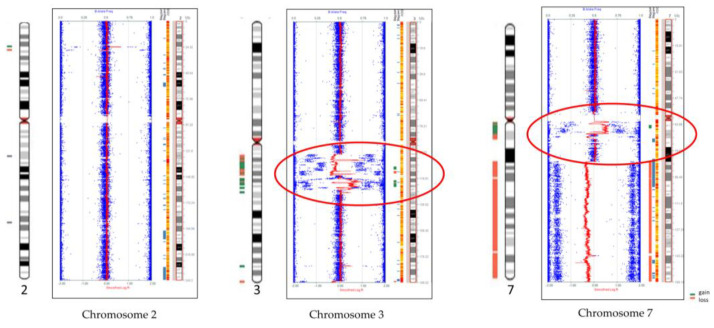
Genome-wide SNP microarray revealed chromoanagenesis regions on chromosomes 3 and 7 (red circles) and loss of 7q (7q21.11−7q26.3). Chromosome 2 is normal except for two small losses and one gain. Blue dots for genotype (B allele frequency) and red lines for copy number based on probe intensities.

Genome-wide SNP microarray analysis revealed nine gains, twenty losses, and three chromoanagenesis regions at 3q12.2–q13.31, 3q13.32–q21.3, and 7p12.2–q11.2 (Figure 2 and Supplementary Table S2). The patient’s chromoanagenesis regions had multiple gains and losses.

### 3.2. Mate Pair Sequencing

**Figure 3:**
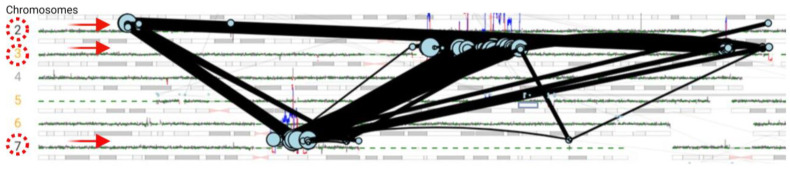
Mate pair sequencing revealed complex rearrangement involving chromosomes 2, 3, and 7. Only structural variants involving these three chromosomes are shown by genome plot (black lines), and breakpoints are shown by solid light green circles. Red arrows pointed to chromosomes 2, 3, and 7.

MPseq revealed 138 SV breakpoints as putative junctions involving chromosomes 2, 3, and 7, which included 69 regions for chromosome 3, 41 for chromosome 7, and 18 for chromosome 2 (Figure 3, Supplementary Table S3). SVs occurred on the p arm of chromosome 2, on the q arm of chromosome 3, and on the surrounding regions of the centromere of chromosome 7. These SVs lead to 16 novel gene fusions and 33 truncated genes (Supplementary Table S3).

Besides these three chromosomes, MPseq also revealed SVs involving chromosomes 1, 5, 6, 16, 17, 20, and 22 (Supplementary Figure S1).

### 3.3. SVs by Nanopore Sequencing

SVs were analyzed based on the CRISPR/Cas9 crRNA sequences (Supplemental Table S1) in this study, as well as all SVs involving breakpoints on chromosomes 2, 3, and 7. We designed thirty CRISPR/Cas9 crRNA sequences to characterize three chromoanagenesis regions detected by SNP microarray including two losses and ten gains of two chromoanagenesis regions on chromosome 3 as well as two losses and four gains of chromoanagenesis regions on chromosome 7 (Supplementary Table S1).

#### 3.3.1. SVs Based on CRISPR/Cas9 crRNA Sequences

**Figure 4:**
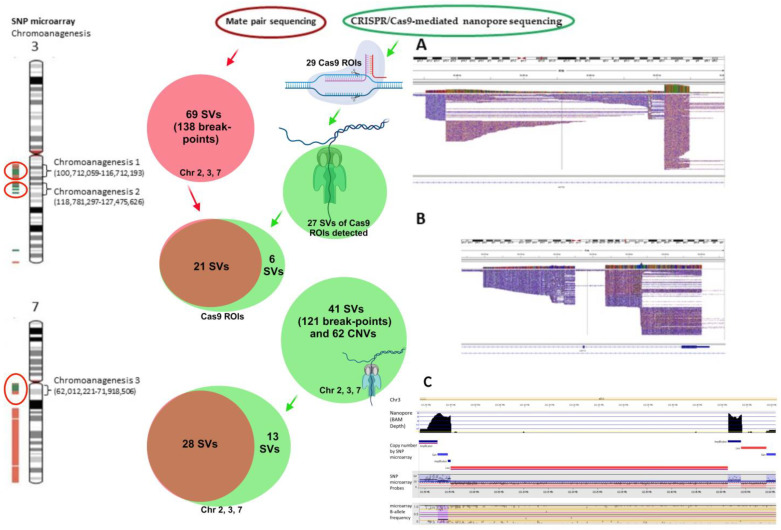
Summarization of various structural variant breakpoints revealed by CRISPR/Cas9-mediated nanopore sequencing, MPseq, and SNP microarray. From left to right, SNP microarray revealed three chromoanagenesis regions: two on chromosome 3q and one on 7q (shown by red circles). Mate pair sequencing data is in a solid red circle, CRISPR/Cas9 nanopore sequencing data is in green circles, and overlapped data of MPseq and CRISPR/cas9 are in brown circles. (**A**,**B**) Complex SVs in the IGV view. Reads of chromosome 7q11.22 genomic region (69,431,166–69,480,285, **A**) and 3q21.3 genomic region (126,248,059–126,263,338, **B**). (**C**) Copy number variants by nanopore sequencing and SNP microarray. Reads of chromosome 3q21.1 genomic region (121,889,250–122,611,342) show complex SVs including amplification, gain, and loss detected by nanopore sequencing, which were consistent with SNP microarray findings. Nanopore reads and SNP microarray data were analyzed by the VIA software to generate copy number variants. Chr: chromosome, CNVs: copy number variants, ROIs: regions of interest by CRISPR/cas9 guideRNAs, SVs: structural variants.

For on-target SVs involving chromosomes 3 and 7 based on CRISPR/Cas9 crRNA sequences, 27 of 29 SVs (93.1%) were on-target by nanopore sequencing (Table 1, Figure 4, supplementary Table S4). Of these 27 SVs, 8 mapped to chromosome 7, and 19 mapped to chromosome 3. The two crRNA sequences that failed to detect their respective SVs had one targeted for chromosome 3 and one targeted for chromosome 7.

#### 3.3.2. SVs Involving Chromosomes 2, 3, and 7

Chromosome-wide SV analysis of chromosomes 2, 3, and 7 revealed an additional 14 SVs besides these on-target SVs, revealing a total of 41 SVs (Supplementary Table S4). Of the 41 SVs involving chromosomes 2, 3, and 7, 28 SVs (68.3%) were from chromosome 3, 10 SVs (24.4%) were from chromosome 7, and 3 (7.3%) were from chromosome 2. In addition to simple SVs, complex SVs involving over two breakpoints were also revealed (Figure 4A,B). Twenty-five SVs (61.0%) involved sequences at introns/exons of genes, twelve (29.2%) were at intergenic regions, and four SVs (9.8%) involved sequences at both introns and intergenic regions (Table 1, Supplementary Table S4). Forty-one SVs involved 121 breakpoints (Supplementary Table S4). Furthermore, amplification of centromeric/pericentric regions of chromosome 7 was detected.

### 3.4. Comparison between MPseq and Nanopore Sequencing

For a total of 27 on-target SVs involving chromosomes 3 and 7 detected by CRISPR/Cas9-mediated nanopore sequencing, 21 SVs (77.8%) were detected by MPseq with shared SV breakpoints (Table 2, Figure 4, Supplementary Table S4). The remaining 6 on-target SVs detected by CRISPR/Cas9-mediated nanopore sequencing were located at distal/proximal locations of MPseq breakpoints or had low coverage reads.

Among 41 SVs involving chromosomes 2, 3, and 7 determined by nanopore sequencing, 28 SVs (68.3%) had shared breakpoints as determined by both MPseq and nanopore (Supplementary Table S4). Of the 28 SVs, 19 SVs (67.9%) were mapped to chromosome 3, 8 SVs (28.6%) were mapped to chromosome 7, and 1 SV (3.6%) was mapped to chromosome 2. Nine SVs detected by MPseq and nanopore involved multiple breakpoints.

### 3.5. Copy Number Variant Analysis

CNVs for the 41 SVs involving chromosomes 2, 3, and 7 by nanopore sequencing were analyzed and compared with SNP microarray results using VIA software (Bionano company, San Diego, CA, USA). These SVs had a total of 62 CNVs including 35 gains, 12 amplifications, and 15 losses (Table 2, Supplementary Table S4). Complex CNVs were common (Figure 4C). Gains/amplifications were more frequent than losses. Chromosome 3 had more gains/amplifications (31) than losses (8). Chromosome 7 had 13 gains/amplification and 7 losses. Chromosome 2 had two gains and one amplification. High amplification of the chromosome 7 centromere was found.

### 3.6. DNA Sequences Flanking the SV Breakpoints

**Figure 5:**
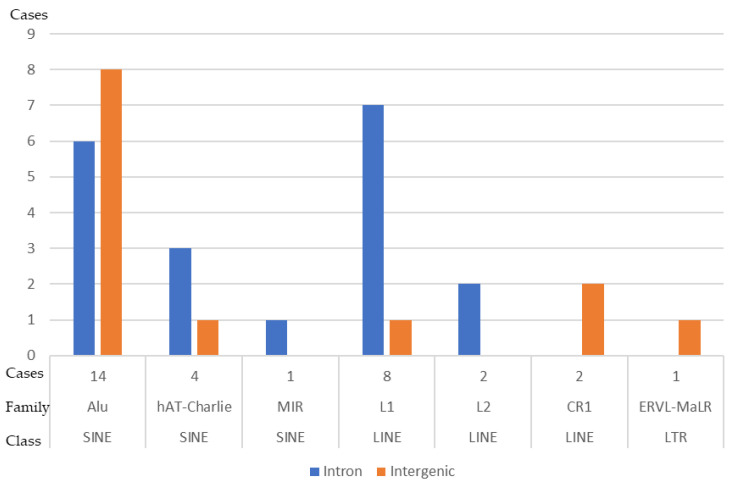
Repeat elements flanking the breakpoints of structural variants in this study. Alu = arthrobactor luteus; CR1 = chicken receptor 1, ERVL = endogenous retrovirus repetitive element, hAT = the hAT superfamily of DNA transposons; LINE = long interspersed nuclear element, L1 = LINE-1, L2 = LINE-2, LTR = long terminal repeat element, MIR = mammalian-wide interspersed repeat, SINE = short interspersed nuclear element.

To understand the genome architecture at SV breakpoints and the role of unusual DNA sequences such as low-copy repeats or tandem repeats [52,53] in chromoanagenesis, we checked for all repeat elements at the SV breakpoints using RepeatMasker [http://www.repeatmasker.org, 15 August 2023] and Repbase update programs [54]. Of the 55 SV breakpoints that were detected by MPseq and had sequencing reads by nanopore sequencing, 19 were intergenic, 35 were at intronic regions, and 1 was at an exon (Supplemental Table S5). A variety of repeats were detected in 32 out of 55 breakpoints (58.2%), including short interspersed nuclear elements (SINEs, a total of 19), long interspersed nuclear elements (LINEs, a total of 12), and long terminal repeat elements (LTRs, a total of 1) (Figure 5).

Of the 19 breakpoints that were intergenic (9 for chromosome 3 and 10 for chromosome 7), 16 had no significant motifs to note. The remaining three off-gene breakpoints had repeat motifs (L3, L1ME1, and L1MEg/Charlie1a, with the former being on chromosome 7 and the latter two being on chromosome 7). Of the 35 mapped to an intronic region, 6 breakpoints lie on gene regions that have repeated motifs. Two breakpoints came from chromosome 2 on the intron of the gene *ATAD2B* and had L1MEa repeat motifs; two breakpoints on chromosome 3 on the introns of the genes *CFAP91* and *SEMA5B* had repeat motifs of L1ME4a, and HAL1, respectively. The remaining two breakpoints lie on chromosome 7 on the intron of the gene *POM121* and have L1MEc and L1MB4 repeat motifs. Of the 35 intronic breakpoints, 17 (14 from chromosome 3 and 3 from chromosome 7) lie on non-repetitive regions of the genes *ATP6V1A* (4), *LSAMP* (4), *MGLL* (3), *CBLB* (1), *CASR* (1), *ROPN1B* (1), and *AUTS2* (3). Of the 35 intronic breakpoints, 11 (9 from chromosome 3 and 2 from chromosome 7) lie on gene regions with and without repeat motifs: *SLC49A4* (4), *ALDH1L1* (2), *CHCHD6* (3), and *GLANT17* (2). One of three regions on the intron of the gene *SLC49A4* had a repeat motif (L2a), one of two regions on the intron of the gene *ALDH1L1* had a repeat motif (L1MB1), two of three regions on the intron of the gene *CHCHD6* had a repeat motif (L1MB7), and one of two regions on the intron of the gene *GALNT17* had a repeat motif (MER5A). Two breakpoints on gene *CHST13* lie on an intron and an exon, with neither having repeat motifs.

### 3.7. NGS Gene Mutation Panel

The NGS gene mutation panel revealed a homozygous mutation in the *TP53* gene (chr17:7574034 C>T; c.994-1G>A) and a *DNMT3A* mutation (chr2:25464543 A>C; p.V657G). The DNMT3A p.V657G mutation is in the protein’s DNA methylase domain. In vitro studies showed that the p.V657G mutation led to DNMT3A inactivation by reduced methyltransferase function and protein stability [55].

## 4. Discussion

Although complex structural chromosome abnormalities (chromoanagenesis) have been reported in AML/MDS, this is the first study using CRISPR/Cas9-mediated nanopore sequencing, MPseq, and SNP microarray analysis along with classic cytogenetic methods (conventional chromosome analysis and FISH) to characterize chromoanagenesis events involving chromosomes 2, 3, and 7. The complex chromoanagenesis events in this study not only have multiple gains and a few losses involving chromosomes 2, 3, and 7, but also have amplification of the chromosome 7 centromere and a pseudotricentric chromosome 7. A pseudotricentric chromosome is a tricentric structure in which only one centromere is active. Chromoanagenesis events along with amplification of the chromosome 7 centromere and a pseudotricentric chromosome 7 have not been reported previously. Furthermore, the presence of centromeric repetitive sequences among chromoanagenesis events adds an extra challenge in characterizing and mapping these SV breakpoints.

Unlike NGS and WGS, CRISPR/Cas9-mediated nanopore sequencing allows the enrichment of genomic regions of interest without PCR amplification, which eliminates potential strand biases due to PCR amplification [42]. Furthermore, when short-read NGS and WGS are used, it is usually hard to have good coverage of repeat sequences, especially centromeric regions [35]. In this study, the amplification of chromosome 7 centromeric regions was detected by long-read nanopore sequencing. Centromeres are vital for genetic stability and inheritance [56]. Research on centromeres is limited, as they are not typically studied [57,58,59]. Although complex involvement of chromosome 7 centromeric regions in chromoanagenesis has not been reported in AML/MDS, studies in the Cryptococcus species demonstrated that multiple DNA double-strand breaks (DSBs) at centromere-specific retrotransposons can lead to the formation of multiple interchromosomal rearrangements (chromothripsis-like events) [60].

Our CRISPR/Cas9 crRNAs were designed for the detection of chromoanagenesis events as revealed by SNP microarray analysis. The sequence of the guideRNA recognizes the adjacent sequence of our genomic region of interest, where endonuclease activity occurs on the recognized sequence. The resulting genomic regions of interest are then examined using nanopore sequencing. Besides the high percentage of on-target SVs (93.3%), additional SVs involving chromosomes 2, 3, and 7 were detected, some of which are consistent with MPseq data. As a whole-genome approach, it is not surprising that MPseq revealed more SVs compared to a targeted approach by CRISPR/Cas9-mediated nanopore sequencing.

Although multiple mechanisms were previously proposed for rearrangements of the complex genomic structure (chromoanagenesis), chromothripsis followed by NHEJ repair may have implications in this study. The chromoanagenesis event in our patient involves chromosome 3 as revealed by SNP microarray analysis. MPseq and targeted nanopore sequencing using a CRISPR/Cas9 approach further characterize this chromoanagenesis event involving multiple SVs and CNVs of chromosomes 2, 3, and 7, which leads us to speculate that our patient’s chromoanagenesis event involves rearrangement of the genomic structure driven by chromothripsis and repaired through NHEJ following extensive DSBs. During NHEJ, small amounts of DNA are removed during the processing phase before being ligated together randomly through DNA ligase. Several DSBs rejoined randomly would result in improper DNA repair and cause translocation of genetic material or rearrangement of the genomic structure that could lead to disruptions of tumor suppressors or amplification of oncogenes. This could explain the patient’s observed translocations/rearrangements among chromosomes 2, 3, and 7, and the loss of 7q genetic material forming a derivative chromosome 7.

Chromothripsis is not very rare in AML and is commonly associated with derivative, marker, and ring chromosomes. Chromothripsis in AML has been reported to influence patient prognosis and disease biology. It has been detected on various chromosomes such as 3, 5, 6, 7, 8, 10, 11, 12, 15, 17, and 20, with the most affected chromosomes being 12, 17, and 5 [31]. Besides gene fusions [61], segmental deletions are common, which include deletions of regions/genes 4q28–4q32 (*SFRP2*), 7q31.1–7q36.3 (*CAV1*, *EPHA1*, and *NRF1*), 12p11.21–12p13.3 (*EPS8*, *RECQL*, and *GUCY2C*), 16q22–16q24.3 (*CBFA2T3*, *FOXF1*, *CDT1*, and *FANCA*), and 17p13–17p13.1 (*ALOX12* and *CLDN7*), with the 5q31.1–5q33.1 deletion noted to be the most frequent [31]. The reported genes most often involved in a chromothripsis event are the amplification of *MYC* on 8q24 and *KMT2A* on 11q23 (most common) [31]. AML with deleted 5q or loss of *TP53/*mutations or deletions of 17q are frequent with chromothripsis [62]. The presence of a 7q deletion including 7q31.1–36.3 and homozygous *TP53/*mutations in our AML patient suggests the involvement of a chromothripsis event forming a highly complex derivative chromosome 7.

Given the high amplification of the chromosome 7 centromere, the gain of extra chromosome 7 centromere sequences (tricentric), and multiple gains of genomic material (mainly involving chromosomes 3 and 7), as found in this case, chromoanasynthesis via FoSTes/MMBIR joining [1,2,63] could be another potential mechanism responsible for the formation of the complex derivative chromosome 7. Chromoanasynthesis occurs through DNA replication error, and template switching through FoSTes or MMBIR occurs at a replication fork, forcing replication to use a template of a nearby sequence or chromosome in the nucleus [2]. Frequent template switches result in complex rearrangements and re-start replication forks.

Recurrent genetic abnormalities including SVs, especially gene fusions, gene rearrangements, and CNVs are important in aiding AML diagnosis and classification, as well as providing information about the prognosis [30]. In general, current clinical genetic diagnostic methods (such as karyotype, FISH, SNP microarray, and short-read-based NGS assays) are incapable of providing high-resolution characterization of SVs and CNVs. Chromoanagenesis contributes to the formation and development of cancer via massive SVs and CNVs, which may disrupt the activity of tumor suppressor genes, activate oncogenes, and/or generate fusion proteins with oncogenic potential. Oncogenic SVs and CNVs along with mutations (such as *TP53*) promote the survival of cancer cells with massive genetic abnormalities. The identification of SVs and CNVs of chromoanagenesis may be useful for further classifying distinct subtypes in myeloid malignancies. We postulate that these subtypes in the future may be defined by the genomic composition of chromoanagenesis, SVs, CNVs, and mutational status. Comprehensive characterization of SVs and CNVs not only provides insights into the underlying molecular mechanisms of cancer development and may advance further classification of AML subtypes, but also may contribute to the identification of new therapeutic targets and the development of innovative treatment approaches. Frequently, targeted therapies designed to inhibit the activity of specific genes or fusion proteins can be more specific and less toxic than traditional chemotherapy.

In this case, complex chromosomal rearrangements lead to gene fusions, gene rearrangements, truncated genes, gain/amplification of genes, and loss of genes. The genes involving SVs in this study are commonly associated with the nervous system [such as *AUTS2* (OMIM*607270), *DPYSL5* (OMIM*608383), *LSAMP* (OMIM*603241), *STXBP5L* (OMIM*609381)], immune system [such as *CD86* (OMIM*601020)], and various cellular functions, along with novel genes. A few cancers or cancer-related genes [such as *EPHA1* (OMIM*179610), *MGLL* (OMIM*609699), *DLG1* (OMIM*601014), *SLC49A4* (OMIM *602773)] have also been observed. *EPHA1* (OMIM*179610) is a receptor tyrosine kinase gene, and overexpression of oncogene EPHA1 was found in hepatoma and lung cancer [64,65]. Elevated MGLL has been described in aggressive human cancer cells [66]. Although *PPP1R2::DLG1* and truncated *DLG1* found in this case have not been reported in AML, *DLG1* has been suggested to play a role in cell proliferation control, like tumor suppressor genes [67,68]. A translocation breakpoint involving *SLC49A4* has been associated with a familial renal cell carcinoma [69].

Breakpoints of complex chromosomal rearrangements in this study are more frequent in genes compared to intergenic regions. Breakpoints at intronic gene regions seem to be more frequent than breakpoints at exons. Over half of these breakpoints are associated with known repeat elements. It is well known that points of genomic instability can be generated by these repetitive sequences, and these repeat elements may serve as substrates for complex structural rearrangements [70,71]. Both LINE sequences and Alu repeats at SV breakpoints are frequent in this case. LINE-1 (L1) is a well-known endogenous mutagen with both DNA endonuclease [72] and reverse transcriptase activities [73]. L1 can mobilize not only itself [74,75], but also other retrotransposons such as Alu [76,77]. Somatic (tissue-specific) non-allelic recombination between homologous repetitive elements contributes to human diseases. Centromeres and cancer-associated genes are enriched for retroelements that may act as recombination hotspots [78]. Retroelement recombination may lead to genomic instability, structural variants, and segmental duplications [35,78,79,80,81,82]. Widespread somatic recombination of L1 and Alu elements may serve as potential mutagens in the genome [78,83]. The abundance of L1 and Alu elements at SV breakpoints in our patient may suggest active and inactive retrotransposons involving a chromoanagenesis event. Non-allelic recombination between homologous repetitive elements involving the chromosome 7 centromere and cancer-associated genes may play a role in the formation of this complex derivative chromosome 7. Further studies of SV breakpoint junctions involved in AML chromoanagenesis cases will be necessary to elucidate the role of these endogenous mutagens in chromoanagenesis formation.

Our combination approach serves to characterize the mechanism of this chromoanagenesis event. While this approach is beneficial, there are still some drawbacks, one of which is the accuracy of long-read sequencing. While long reads provide the benefit of better human genome understanding and the ability to access unreadable regions from NGS, their accuracy, cost, and efficiency provide limitations with their usage. Compared to short-read NGS, the accuracy of long reads is low and variable in certain situations [84,85,86,87]. This inconsistency in accuracy produces challenges in gene annotations and the complete understanding of a genome [88]. However, with polishing tools, the accuracy is improved, although most polishing tools require a reference and, in some cases, are dependent on the short-read sequences of the individual [89,90]. This leads to another challenge that long reads pose, their cost and efficiency. Compared to WGS through short-read sequencing, WGS through long-read sequencing is overall expensive and time-consuming, where in some cases, it could take weeks to obtain results [84,85,91]. Therefore, further advanced analysis software of long-read sequencing is needed to provide fast personalized oncogenomics in a single sequencing assay (such as nanopore sequencing of native DNA without PCR amplification) to detect large, complex SVs, CNVs, and potential epigenetic modifications via genomic phasing using haplotype-specific methylation calls [92].

In this proof-of-principle study, we demonstrated the feasibility of this integrated approach in an AML patient carrying three chromoanagenesis events. Given the rarity of chromoanagenesis in hematological malignancies and the lack of well-characterized chromoanagenesis events in commonly available cell lines or accessible specimens of cancer patients, the major limitation of this study is a single AML case. While this study identified SVs and CNVs of chromoanagenesis that may be useful for further classifying distinct subtypes in myeloid malignancies, comprehensive studies of the different myeloid subtypes’ chromoanagenesis, SVs, CNVs, and molecular profiles and their impact on disease outcomes are needed to inform clinical decision making. Future studies that accumulate more well-characterized chromoanagenesis from multiple centers, obtain comprehensive clinical data, and follow various treatment strategies in patients with myeloid malignancies will shed light on treatment response rates, survival rates, and overall prognosis of these patients.

## 5. Conclusions

To our knowledge, our case is the first case with complex chromoanagenesis involving chromosomes 2, 3, and 7 along with a pseudotricentric chromosome 7 centromere and amplification of the chromosome 7 centromere. This report emphasizes the value of performing an integrated approach including long-read nanopore sequencing, MPseq, and cytogenomic methods to characterize complex structural rearrangements in AML. The long reads from the nanopore not only determined simple structural abnormalities but also enabled us to resolve the long-range structure of the complex chromoanagenesis. Sequencing the cancer genome of our patient using CRISPR/Cas9-mediated targeted sequencing on nanopore results detected breakpoints of complex structural chromosome abnormalities that are highly sensitive. The characterization of these complex structural chromosome abnormalities not only will help understand the molecular mechanisms responsible for the formation and development of chromoanagenesis, but also may identify specific molecular targets and their impact on therapy and overall survival. This combination approach in the characterization of chromoanagenesis and other structural abnormalities may be useful for both clinical and research applications.

## Figures and Tables

**Table 1:** Structural variants by CRISPR/Cas9-mediated nanopore sequencing.

ID	Chromo-some	Structural Variant (SV) Type	crRNA #	Copy Number Variant (CNV)	Genomic Location (Exon, Intron, Non-Gene Regions)
1	3	Simple	1	Gain	intron (*ABI3BP*)
2	3	Complex	2	Gain, amplification	intergenic region
3	3	Complex	3	Gain, amplification	intron *(CBLB*)
4	3	Complex	4	Loss, gains	exon (*USF3*), intergenic
5	3	Simple	5	Gain, loss	intron (*ATP6V1A*)
6	3	Simple	6	Loss, gain, amplification	intron (*LSAMP*)
7	3	Complex	7	Gains, loss	intron (*LSAMP*)
8	3	Complex	8	Normal	intergenic region
9	3	Complex	9	Normal	intron (*NR_135547.1*)
10	3	Complex	11	Gain	intron (*CFAP91*), intergenic
11	3	Complex	12	Gains, loss	intron (CASR), intergenic
12	3	Complex	13	Gain	intron (*SLC49A4*), exon and intron (*SEMA5B*), intergenic
13	3	Complex	14	Gain, amplification, normal	intron (*ALG1L*)
14	3	Complex	15	Gain, amplification	intron (*ALD1H1*)
15	3	Simple	16	Normal	intron (*ALD1H1*)
16	3	Simple	17	Normal	intron (*CHST13*)
17	3	Complex	18	Normal	exon and intron (*CHST13*)
18	3	Simple	19	Gain	intron (*CHCHD6*)
19	3	Complex	20	Gain	intron (*PODXL2*), intron (*MGLL*)
20	7	Complex	21	Gain, amplification, normal	intergenic region
21	7	Complex	23	Loss, gains	intergenic region
22	7	Complex	24	Gain	intergenic region
23	7	Simple	25	Gain	intergenic region
24	7	Complex	26	Gain	intron (*AUTS2*)
25	7	Complex	27	Gain	intron (*AUTS2*)
26	7	Simple	28/29	Gain, loss	intron (*AUTS2*)
27	7	Complex	30	Loss, normal	intron (*GALNT17*)

**Table 2:** Comparison of data of SNP microarray, MPseq, and CRISPR/Cas9-mediated nanopore sequencing.

Chromosome Bands	Genomic Regions	SNP Microarray	Mate Pair Sequencing	CRISPR/Cas9 Nanopore Sequencing	
				Structural Variant (SV)	Copy Number Variant (CNV)
Chromoanagenesis					
3q12.2–q13.31	chr3:100712059-116712193	2 losses, 3 gains	13 breakpoints	27 breakpoints	6 losses, 9 gains, 3 amplification
3q13.32–q22.1	chr3:118281297-129837325	7 gains	47 breakpoints	50 breakpoints	2 losses, 13 gains, 6 amplification
7p12.2–q21.11	chr7:62012221-71918506	2 losses, 4 gains	40 breakpoints	31 breakpoints	7 losses, 11 gains, 2 amplification
Other genomic regions that involved in chromosomes 2, 3, and 7					
2p23.3–p16.3	chr2:24066341-52227175	2 losses, 1 gain	17 breakpoints	4 breakpoints	1 gain, 1 amplification
3q27.2–q29	chr3:185359470-196944509	1 loss, 1 gain	19 breakpoints	none	none
Others	various genomic regions	none	2 breakpoints	9 breakpoints	1 gain
Total		4 losses, 13 gains	138 breakpoints (69 SVs)	121 breakpoints (41 SVs)	62 CNVs (15 losses, 35 gains, 12 amplifications)

Genomic locations are based on the hg19 (GRCh37) genome assembly.

## Data Availability

All requests for primary data and experimental reagents should be addressed to yzou19@jhmi.edu.

## Supplementary Materials

The following supporting information can be downloaded at: https://www.mdpi.com/article/10.3390/biomedicines12030598/s1, Supplementary Table S1: The crRNA sequences used in this study. Supplementary Table S2: Genome-wide SNP microarray data. Supplementary Table S3: Mate pair sequencing data involving chromosomes 2, 3, and 7. Supplementary Table S4: Structural variants involving chromosomes 2, 3, and 7 by nanopore sequencing. Supplementary Table S5: DNA sequences flanking breakpoints of structural variants. Supplementary Figure S1: Genome-wide mate pair sequencing data.

## Abbreviations

alt-EJ	Alternative end joining
AML	Acute myeloid leukemia
ALL	Acute lymphoblastic leukemia
Cas9	CRISPR-associated Protein 9
CIN	Chromosome instability
CNevents	Copy number events
CNs	Copy numbers
CNV	Copy number variants
CRISPR	Clustered regularly interspaced short palindromic repeats
DSBs	Double-strand breaks
DNA	Deoxyribonucleic acid
FAB	French–American–British
FISH	Fluorescence in situ hybridization
FoSTeS	Fork stalling and template switching
guideRNA	Guide ribonucleic acid
IDT	Integrated DNA Technologies
IGV	Integrative Genomics Viewer
LINE	Long interspersed nuclear elements
MDS	Myelodysplastic syndrome
MMBIR	Microhomology-mediated break-induced relocation
MPseq	Mate pair sequencing
NGS	Next-generation sequencing
NHEJ	Non-homologous end joining
PCR	Polymerase chain reaction
RNA	Ribonucleic acid
SOB	Shortness of breath
SNP	Single-nucleotide polymorphism
SHH-MB	Sonic Hedgehog-induced medulloblastoma
SVs	Structural variants
UCSC	University of California Santa Cruz
VIA	Variant Intelligence Applications™
WGS	Whole-genome sequencing

## References

[1] Holland A.J., Cleveland D.W. (2012). Chromoanagenesis and cancer: Mechanisms and consequences of localized, complex chromosomal rearrangements. Nat. Med..

[2] Pellestor F. (2019). Chromoanagenesis: Cataclysms behind complex chromosomal rearrangements. Mol. Cytogenet..

[3] Stephens P.J., Greenman C.D., Fu B., Yang F., Bignell G.R., Mudie L.J., Pleasance E.D., Lau K.W., Beare D., Stebbings L.A. (2011). Massive genomic rearrangement acquired in a single catastrophic event during cancer development. Cell.

[4] Maher C.A., Wilson R.K. (2012). Chromothripsis and human disease: Piecing together the shattering process. Cell.

[5] Korbel J.O., Campbell P.J. (2013). Criteria for inference of chromothripsis in cancer genomes. Cell.

[6] Iliakis G., Murmann T., Soni A. (2015). Alternative end-joining repair pathways are the ultimate backup for abrogated classical non-homologous end-joining and homologous recombination repair: Implications for the formation of chromosome translocations. Mutat. Res. Genet. Toxicol. Environ. Mutagen..

[7] Masset H., Hestand M.S., Van Esch H., Kleinfinger P., Plaisancie J., Afenjar A., Molignier R., Schluth-Bolard C., Sanlaville D., Vermeesch J.R. (2016). A Distinct Class of Chromoanagenesis Events Characterized by Focal Copy Number Gains. Hum. Mutat..

[8] So A., Le Guen T., Lopez B.S., Guirouilh-Barbat J. (2017). Genomic rearrangements induced by unscheduled DNA double strand breaks in somatic mammalian cells. FEBS J..

[9] Liu P., Erez A., Nagamani S.C., Dhar S.U., Kolodziejska K.E., Dharmadhikari A.V., Cooper M.L., Wiszniewska J., Zhang F., Withers M.A. (2011). Chromosome catastrophes involve replication mechanisms generating complex genomic rearrangements. Cell.

[10] Lee J.A., Carvalho C.M., Lupski J.R. (2007). A DNA replication mechanism for generating nonrecurrent rearrangements associated with genomic disorders. Cell.

[11] Branzei D., Foiani M. (2005). The DNA damage response during DNA replication. Curr. Opin. Cell. Biol..

[12] Venkatesan S., Natarajan A.T., Hande M.P. (2015). Chromosomal instability--mechanisms and consequences. Mutat. Res. Genet. Toxicol. Environ. Mutagen..

[13] Hastings P.J., Ira G., Lupski J.R. (2009). A microhomology-mediated break-induced replication model for the origin of human copy number variation. PLoS Genet..

[14] Baca S.C., Prandi D., Lawrence M.S., Mosquera J.M., Romanel A., Drier Y., Park K., Kitabayashi N., MacDonald T.Y., Ghandi M. (2013). Punctuated evolution of prostate cancer genomes. Cell.

[15] Kloosterman W.P., Hoogstraat M., Paling O., Tavakoli-Yaraki M., Renkens I., Vermaat J.S., van Roosmalen M.J., van Lieshout S., Nijman I.J., Roessingh W. (2011). Chromothripsis is a common mechanism driving genomic rearrangements in primary and metastatic colorectal cancer. Genome Biol..

[16] Rausch T., Jones D.T., Zapatka M., Stutz A.M., Zichner T., Weischenfeldt J., Jager N., Remke M., Shih D., Northcott P.A. (2012). Genome sequencing of pediatric medulloblastoma links catastrophic DNA rearrangements with TP53 mutations. Cell.

[17] Zhang J., Ding L., Holmfeldt L., Wu G., Heatley S.L., Payne-Turner D., Easton J., Chen X., Wang J., Rusch M. (2012). The genetic basis of early T-cell precursor acute lymphoblastic leukaemia. Nature.

[18] Wu C., Wyatt A.W., McPherson A., Lin D., McConeghy B.J., Mo F., Shukin R., Lapuk A.V., Jones S.J., Zhao Y. (2012). Poly-gene fusion transcripts and chromothripsis in prostate cancer. Genes Chromosomes Cancer.

[19] Northcott P.A., Shih D.J., Peacock J., Garzia L., Morrissy A.S., Zichner T., Stutz A.M., Korshunov A., Reimand J., Schumacher S.E. (2012). Subgroup-specific structural variation across 1,000 medulloblastoma genomes. Nature.

[20] Magrangeas F., Avet-Loiseau H., Munshi N.C., Minvielle S. (2011). Chromothripsis identifies a rare and aggressive entity among newly diagnosed multiple myeloma patients. Blood.

[21] Molenaar J.J., Koster J., Zwijnenburg D.A., van Sluis P., Valentijn L.J., van der Ploeg I., Hamdi M., van Nes J., Westerman B.A., van Arkel J. (2012). Sequencing of neuroblastoma identifies chromothripsis and defects in neuritogenesis genes. Nature.

[22] Wahl G.M. (1989). The importance of circular DNA in mammalian gene amplification. Cancer Res..

[23] Kou F., Wu L., Ren X., Yang L. (2020). Chromosome Abnormalities: New Insights into Their Clinical Significance in Cancer. Mol. Ther. Oncolytics.

[24] Holland A.J., Cleveland D.W. (2009). Boveri revisited: Chromosomal instability, aneuploidy and tumorigenesis. Nat. Rev. Mol. Cell. Biol..

[25] Weaver B.A., Cleveland D.W. (2008). The aneuploidy paradox in cell growth and tumorigenesis. Cancer Cell.

[26] Ly P., Brunner S.F., Shoshani O., Kim D.H., Lan W., Pyntikova T., Flanagan A.M., Behjati S., Page D.C., Campbell P.J. (2019). Chromosome segregation errors generate a diverse spectrum of simple and complex genomic rearrangements. Nat. Genet..

[27] Janssen A., van der Burg M., Szuhai K., Kops G.J., Medema R.H. (2011). Chromosome segregation errors as a cause of DNA damage and structural chromosome aberrations. Science.

[28] Li Y., Roberts N.D., Wala J.A., Shapira O., Schumacher S.E., Kumar K., Khurana E., Waszak S., Korbel J.O., Haber J.E. (2020). Patterns of somatic structural variation in human cancer genomes. Nature.

[29] Taylor A.M., Shih J., Ha G., Gao G.F., Zhang X., Berger A.C., Schumacher S.E., Wang C., Hu H., Liu J. (2018). Genomic and Functional Approaches to Understanding Cancer Aneuploidy. Cancer Cell.

[30] Sandberg A.A. (1991). Chromosome abnormalities in human cancer and leukemia. Mutat. Res..

[31] Fontana M.C., Marconi G., Feenstra J.D.M., Fonzi E., Papayannidis C., Ghelli Luserna di Rora A., Padella A., Solli V., Franchini E., Ottaviani E. (2018). Chromothripsis in acute myeloid leukemia: Biological features and impact on survival. Leukemia.

[32] Gao J., Chen Y.H., Mina A., Altman J.K., Kim K.Y., Zhang Y., Lu X., Jennings L., Sukhanova M. (2020). Unique morphologic and genetic characteristics of acute myeloid leukemia with chromothripsis: A clinicopathologic study from a single institution. Hum. Pathol..

[33] MacKinnon R.N. (2018). Analysis of Chromothripsis by Combined FISH and Microarray Analysis. Methods Mol. Biol..

[34] Boyd R.J., Murry J.B., Morsberger L.A., Klausner M., Chen S., Gocke C.D., McCallion A.S., Zou Y.S. (2023). Ring Chromosomes in Hematological Malignancies Are Associated with *TP53* Gene Mutations and Characteristic Copy Number Variants. Cancers.

[35] Bailey J.A., Yavor A.M., Massa H.F., Trask B.J., Eichler E.E. (2001). Segmental duplications: Organization and impact within the current human genome project assembly. Genome Res..

[36] Sudmant P.H., Rausch T., Gardner E.J., Handsaker R.E., Abyzov A., Huddleston J., Zhang Y., Ye K., Jun G., Fritz M.H. (2015). An integrated map of structural variation in 2,504 human genomes. Nature.

[37] Hodgkinson A., Chen Y., Eyre-Walker A. (2012). The large-scale distribution of somatic mutations in cancer genomes. Hum. Mutat..

[38] Hills M., Jeyapalan J.N., Foxon J.L., Royle N.J. (2007). Mutation mechanisms that underlie turnover of a human telomere-adjacent segmental duplication containing an unstable minisatellite. Genomics.

[39] Hastings P.J., Lupski J.R., Rosenberg S.M., Ira G. (2009). Mechanisms of change in gene copy number. Nat. Rev. Genet..

[40] Pollard M.O., Gurdasani D., Mentzer A.J., Porter T., Sandhu M.S. (2018). Long reads: Their purpose and place. Hum. Mol. Genet..

[41] Mantere T., Kersten S., Hoischen A. (2019). Long-Read Sequencing Emerging in Medical Genetics. Front. Genet..

[42] Gilpatrick T., Lee I., Graham J.E., Raimondeau E., Bowen R., Heron A., Downs B., Sukumar S., Sedlazeck F.J., Timp W. (2020). Targeted nanopore sequencing with Cas9-guided adapter ligation. Nat. Biotechnol..

[43] Pitel B.A., Zuckerman E.Z., Baughn L.B. (2023). Mate Pair Sequencing: Next-Generation Sequencing for Structural Variant Detection. Methods Mol. Biol..

[44] Smadbeck J., Peterson J.F., Pearce K.E., Pitel B.A., Figueroa A.L., Timm M., Jevremovic D., Shi M., Stewart A.K., Braggio E. (2019). Mate pair sequencing outperforms fluorescence in situ hybridization in the genomic characterization of multiple myeloma. Blood Cancer J..

[45] Smadbeck J.B., Johnson S.H., Smoley S.A., Gaitatzes A., Drucker T.M., Zenka R.M., Kosari F., Murphy S.J., Hoppman N., Aypar U. (2018). Copy number variant analysis using genome-wide mate-pair sequencing. Genes Chromosomes Cancer.

[46] Johnson S.H., Smadbeck J.B., Smoley S.A., Gaitatzes A., Murphy S.J., Harris F.R., Drucker T.M., Zenka R.M., Pitel B.A., Rowsey R.A. (2018). SVAtools for junction detection of genome-wide chromosomal rearrangements by mate-pair sequencing (MPseq). Cancer Genet..

[47] Drucker T.M., Johnson S.H., Murphy S.J., Cradic K.W., Therneau T.M., Vasmatzis G. (2014). BIMA V3: An aligner customized for mate pair library sequencing. Bioinformatics.

[48] Jiang L., Pallavajjala A., Huang J., Haley L., Morsberger L., Stinnett V., Hardy M., Park R., Ament C., Finch A. (2021). Clinical Utility of Targeted Next-Generation Sequencing Assay to Detect Copy Number Variants Associated with Myelodysplastic Syndrome in Myeloid Malignancies. J. Mol. Diagn..

[49] Pallavajjala A., Haley L., Stinnett V., Adams E., Pallavajjala R., Huang J., Morsberger L.A., Hardy M., Long P., Gocke C.D. (2022). Utility of targeted next-generation sequencing assay to detect 1p/19q co-deletion in formalin-fixed paraffin-embedded glioma specimens. Hum. Pathol..

[50] Adzhubei I.A., Schmidt S., Peshkin L., Ramensky V.E., Gerasimova A., Bork P., Kondrashov A.S., Sunyaev S.R. (2010). A method and server for predicting damaging missense mutations. Nat. Methods.

[51] Sim N.L., Kumar P., Hu J., Henikoff S., Schneider G., Ng P.C. (2012). SIFT web server: Predicting effects of amino acid substitutions on proteins. Nucleic Acids Res..

[52] Smit A.F. (1999). Interspersed repeats and other mementos of transposable elements in mammalian genomes. Curr. Opin. Genet. Dev..

[53] Smit A.F. (1996). The origin of interspersed repeats in the human genome. Curr. Opin. Genet. Dev..

[54] Jurka J. (2000). Repbase update: A database and an electronic journal of repetitive elements. Trends Genet..

[55] Huang Y.H., Chen C.W., Sundaramurthy V., Slabicki M., Hao D., Watson C.J., Tovy A., Reyes J.M., Dakhova O., Crovetti B.R. (2022). Systematic Profiling of DNMT3A Variants Reveals Protein Instability Mediated by the DCAF8 E3 Ubiquitin Ligase Adaptor. Cancer Discov..

[56] Mackinnon R.N., Campbell L.J. (2013). Chromothripsis under the microscope: A cytogenetic perspective of two cases of AML with catastrophic chromosome rearrangement. Cancer Genet..

[57] Mackinnon R.N., Wall M., Zordan A., Nutalapati S., Mercer B., Peverall J., Campbell L.J. (2013). Genome organization and the role of centromeres in evolution of the erythroleukaemia cell line HEL. Evol. Med. Public Health.

[58] Garsed D.W., Marshall O.J., Corbin V.D., Hsu A., Di Stefano L., Schroder J., Li J., Feng Z.P., Kim B.W., Kowarsky M. (2014). The architecture and evolution of cancer neochromosomes. Cancer Cell.

[59] Macchia G., Nord K.H., Zoli M., Purgato S., D’Addabbo P., Whelan C.W., Carbone L., Perini G., Mertens F., Rocchi M. (2015). Ring chromosomes, breakpoint clusters, and neocentromeres in sarcomas. Genes Chromosomes Cancer.

[60] Yadav V., Sun S., Coelho M.A., Heitman J. (2020). Centromere scission drives chromosome shuffling and reproductive isolation. Proc. Natl. Acad. Sci. USA.

[61] Singh Z.N., Richards S., El Chaer F., Duong V.H., Gudipati M.A., Waters E.O., Koon S., Webley M., Pitel B., Hoppman N.L. (2019). Cryptic ETV6-PDGFRB fusion in a highly complex rearrangement of chromosomes 1, 5, and 12 due to a chromothripsis-like event in a myelodysplastic syndrome/myeloproliferative neoplasm. Leuk. Lymphoma.

[62] Rücker F.G., Dolnik A., Blätte T.J., Teleanu V., Ernst A., Thol F., Heuser M., Ganser A., Döhner H., Döhner K. (2018). Chromothripsis is linked to TP53 alteration, cell cycle impairment, and dismal outcome in acute myeloid leukemia with complex karyotype. Haematologica.

[63] Gudipati M.A., Waters E., Greene C., Goel N., Hoppman N.L., Pitel B.A., Webley M.R., Zou Y. (2019). Stable transmission of complex chromosomal rearrangements involving chromosome 1q derived from constitutional chromoanagenesis. Mol. Cytogenet..

[64] Maru Y., Hirai H., Yoshida M.C., Takaku F. (1988). Evolution, expression, and chromosomal location of a novel receptor tyrosine kinase gene, eph. Mol. Cell. Biol..

[65] Kiyokawa E., Takai S., Tanaka M., Iwase T., Suzuki M., Xiang Y.Y., Naito Y., Yamada K., Sugimura H., Kino I. (1994). Overexpression of ERK, an EPH family receptor protein tyrosine kinase, in various human tumors. Cancer Res..

[66] Nomura D.K., Long J.Z., Niessen S., Hoover H.S., Ng S.W., Cravatt B.F. (2010). Monoacylglycerol lipase regulates a fatty acid network that promotes cancer pathogenesis. Cell.

[67] Azim A.C., Knoll J.H., Marfatia S.M., Peel D.J., Bryant P.J., Chishti A.H. (1995). DLG1: Chromosome location of the closest human homologue of the Drosophila discs large tumor suppressor gene. Genomics.

[68] Mori K., Iwao K., Miyoshi Y., Nakagawara A., Kofu K., Akiyama T., Arita N., Hayakawa T., Nakamura Y. (1998). Identification of brain-specific splicing variants of the hDLG1 gene and altered splicing in neuroblastoma cell lines. J. Hum. Genet..

[69] Bodmer D., Eleveld M., Kater-Baats E., Janssen I., Janssen B., Weterman M., Schoenmakers E., Nickerson M., Linehan M., Zbar B. (2002). Disruption of a novel MFS transporter gene, DIRC2, by a familial renal cell carcinoma-associated t(2;3)(q35;q21). Hum. Mol. Genet..

[70] George C.M., Alani E. (2012). Multiple cellular mechanisms prevent chromosomal rearrangements involving repetitive DNA. Crit. Rev. Biochem. Mol. Biol..

[71] Weckselblatt B., Rudd M.K. (2015). Human Structural Variation: Mechanisms of Chromosome Rearrangements. Trends Genet..

[72] Feng Q., Moran J.V., Kazazian H.H., Boeke J.D. (1996). Human L1 retrotransposon encodes a conserved endonuclease required for retrotransposition. Cell.

[73] Mathias S.L., Scott A.F., Kazazian H.H., Boeke J.D., Gabriel A. (1991). Reverse transcriptase encoded by a human transposable element. Science.

[74] Moran J.V., Holmes S.E., Naas T.P., DeBerardinis R.J., Boeke J.D., Kazazian H.H. (1996). High frequency retrotransposition in cultured mammalian cells. Cell.

[75] Kazazian H.H. (2004). Mobile elements: Drivers of genome evolution. Science.

[76] Kajikawa M., Okada N. (2002). LINEs mobilize SINEs in the eel through a shared 3′ sequence. Cell.

[77] Dewannieux M., Esnault C., Heidmann T. (2003). LINE-mediated retrotransposition of marked Alu sequences. Nat. Genet..

[78] Pascarella G., Hon C.C., Hashimoto K., Busch A., Luginbuhl J., Parr C., Hin Yip W., Abe K., Kratz A., Bonetti A. (2022). Recombination of repeat elements generates somatic complexity in human genomes. Cell.

[79] Batzer M.A., Deininger P.L. (2002). Alu repeats and human genomic diversity. Nat. Rev. Genet..

[80] Sen S.K., Han K., Wang J., Lee J., Wang H., Callinan P.A., Dyer M., Cordaux R., Liang P., Batzer M.A. (2006). Human genomic deletions mediated by recombination between Alu elements. Am. J. Hum. Genet..

[81] Lee J., Han K., Meyer T.J., Kim H.S., Batzer M.A. (2008). Chromosomal inversions between human and chimpanzee lineages caused by retrotransposons. PLoS ONE.

[82] Deininger P.L., Batzer M.A. (1999). Alu repeats and human disease. Mol. Genet. Metab..

[83] Nazaryan-Petersen L., Bertelsen B., Bak M., Jonson L., Tommerup N., Hancks D.C., Tumer Z. (2016). Germline Chromothripsis Driven by L1-Mediated Retrotransposition and Alu/Alu Homologous Recombination. Hum. Mutat..

[84] Miga K.H., Koren S., Rhie A., Vollger M.R., Gershman A., Bzikadze A., Brooks S., Howe E., Porubsky D., Logsdon G.A. (2020). Telomere-to-telomere assembly of a complete human X chromosome. Nature.

[85] Jain M., Koren S., Miga K.H., Quick J., Rand A.C., Sasani T.A., Tyson J.R., Beggs A.D., Dilthey A.T., Fiddes I.T. (2018). Nanopore sequencing and assembly of a human genome with ultra-long reads. Nat. Biotechnol..

[86] Shafin K., Pesout T., Lorig-Roach R., Haukness M., Olsen H.E., Bosworth C., Armstrong J., Tigyi K., Maurer N., Koren S. (2020). Nanopore sequencing and the Shasta toolkit enable efficient de novo assembly of eleven human genomes. Nat. Biotechnol..

[87] Miao H., Zhou J., Yang Q., Liang F., Wang D., Ma N., Gao B., Du J., Lin G., Wang K. (2018). Long-read sequencing identified a causal structural variant in an exome-negative case and enabled preimplantation genetic diagnosis. Hereditas.

[88] Gordon D., Huddleston J., Chaisson M.J., Hill C.M., Kronenberg Z.N., Munson K.M., Malig M., Raja A., Fiddes I., Hillier L.W. (2016). Long-read sequence assembly of the gorilla genome. Science.

[89] Walker B.J., Abeel T., Shea T., Priest M., Abouelliel A., Sakthikumar S., Cuomo C.A., Zeng Q., Wortman J., Young S.K. (2014). Pilon: An integrated tool for comprehensive microbial variant detection and genome assembly improvement. PLoS ONE.

[90] Chin C.S., Alexander D.H., Marks P., Klammer A.A., Drake J., Heiner C., Clum A., Copeland A., Huddleston J., Eichler E.E. (2013). Nonhybrid, finished microbial genome assemblies from long-read SMRT sequencing data. Nat. Methods.

[91] Vollger M.R., Logsdon G.A., Audano P.A., Sulovari A., Porubsky D., Peluso P., Wenger A.M., Concepcion G.T., Kronenberg Z.N., Munson K.M. (2020). Improved assembly and variant detection of a haploid human genome using single-molecule, high-fidelity long reads. Ann. Hum. Genet..

[92] Battaglia S., Dong K., Wu J., Chen Z., Najm F.J., Zhang Y., Moore M.M., Hecht V., Shoresh N., Bernstein B.E. (2022). Long-range phasing of dynamic, tissue-specific and allele-specific regulatory elements. Nat. Genet..

