# Supplementary material for: An Integrated Approach Including CRISPR/Cas9-Mediated Nanopore Sequencing, Mate Pair Sequencing, and Cytogenomic Methods to Characterize Complex Structural Rearrangements in Acute Myeloid Leukemia
Source: Biomedicines. 2024 Mar 7;12(3):598. doi: 10.3390/biomedicines12030598 (PMC10968562; doi:10.3390/biomedicines12030598)
Supplement: Supplementary file 1 [file biomedicines-12-00598-s001.zip › biomedicines-2871940-supplementary.pdf]

Supplementary Table S1: The crRNAs sequences used in this study.

ID	IDT #	Position	Strand	Sequence	PAM	Target range location
1	CD.Cas9.DDMP4713.AB	913	+	TACTGACTCAATCTAACATC	TGG	chr3:100598997-100599996
2	CD.Cas9.JLLC0695.AC	629	-	TCAAGACTGTCTGACCGGCT	GGG	chr3:104891716-104892715
3	CD.Cas9.NXBW1374.AA	187	+	ACTAGTTCATAAAAGGCTAC	AGG	chr3:105474196-105475195
4	CD.Cas9.VYHR2733.AA	843	-	CGTTAGGTGCAACTAACACC	AGG	chr3:113371011-113372010
5	CD.Cas9.GBHY8639.AC	753	+	TAATGCAATCGCAAGTCTGG	TGG	chr3:113493648-113494647
6	CD.Cas9.CJVF1523.AA	6	-	CGACAACCCACTACCTGCAA	AGG	chr3:115544892-115545891
7	CD.Cas9.FBLB1643.AA	183	+	CACGATATGGCTCCTTACAC	TGG	chr3:115702434-115703433
8	CD.Cas9.PBGN2758.AH	672	-	CTGATATTTAAACCTACTGC	AGG	chr3:116841289-116842288
9	CD.Cas9.NJCX3562.AA	398	-	CTCATAACTGGACCTATATT	TGG	chr3:118282298-118283297
10	CD.Cas9.KLJK8708.AC	114	+	TGCAATAGGTCTTGACTCAC	AGG	chr3:118527692-118528691
11	CD.Cas9.QFFJ3076.AB	361	-	AACCACAATTGCCTGTACAT	GGG	chr3:119434301-119435300
12	CD.Cas9.STVN5245.AA	674	+	GATTATTTAGGCTAACGTAG	TGG	chr3:121927055-121928054
13	CD.Cas9.ZJGX3218.AA	128	-	AGAGTCGGGCACCATCCTGA	AGG	chr3:122640824-122641823
14	CD.Cas9.PRYS9418.AF	161	+	GACAAAGGCACGGTGCCTGT	AGG	chr3:125681874-125682873
15	CD.Cas9.BKBF0238.AA	245	-	GGACGGGCCTAAGAACCATG	GGG	chr3:125845982-125846981
16	CD.Cas9.QNPR7457.AB	192	+	CAAACCACGATAATTGTGTA	AGG	chr3:125897367-125898366
17	CD.Cas9.KVZG6545.AC	510	-	AGGCACATTTGACCCACCTC	TGG	chr3:126254507-126255506
18	CD.Cas9.HJFP3604.AD	307	+	ATTACGTCAACCCATAAGGT	GGG	chr3:126256356-126257355
19	CD.Cas9.CBWD4698.AA	111	-	CACGGGTTTAAACCACAATA	AGG	chr3:126441249-126442248
20	CD.Cas9.RPJQ0683.AB	957	+	AATCTGCTCCAAGCGTGGAT	GGG	chr3:127406753-127407752
21	CD.Cas9.BFJL6275.AA	124	-	TGGACCTGCAAGTTTGTAGT	TGG	chr7:62013222-62014221
22	CD.Cas9.SPDX4659.AK	817	+	TGGCTCACTCATACCAGGTG	TGG	chr7:62575770-62576769
23	CD.Cas9.VWBZ7809.AF	823	-	TTTAGTTCGGCAATTTCTAC	TGG	chr7:63432015-63433014
24	CD.Cas9.PPVK4627.AA	751	+	TTTATCAAGCCGACATAACA	AGG	chr7:68268995-68269994
25	CD.Cas9.BNVQ1853.AC	429	-	CCATGAGGAAACTCCATTAA	AGG	chr7:68505051-68506050
26	CD.Cas9.XJCJ9825.AA	896	+	GTTCCTATACTGAGATGTGC	TGG	chr7:69242026-69243025
27	CD.Cas9.DSSQ6504.AE	224	-	ACTCCATTTATCAAGGTTAC	AGG	chr7:69438048-69439047
28	CD.Cas9.FHJW9791.AB	236	+	CAAGAAGTGAGTCGTTGCAT	AGG	chr7:69470821-69471820
29	CD.Cas9.NRPK8963.AB	236	+	CAAGAAGTGAGTCGTTGCAT	AGG	chr7:69470821-69471820
30	CD.Cas9.MKWZ0913.AA	600	+	AGGCTGTCGTGTCAACCCAA	AGG	chr7:70992375-70993374

* IDT=Integrated DNA technologies, Coralville, IA, USA. The Target range location is based on the hg19 (GRCh37) genome assembly.

Supplementary Table S2: Genome-wide SNP microarray data.

Bkpts	Abnormality	Start (Approximate)	End (Approximate)	
1pter to p32.3	Gain	1	50,700,000	Terminal
1p32.3	Gain	52,800,000	53,600,000	
1p32.2	Gain	57,700,000	58,600,000	
1p31.3	Gain	63,200,000	64,100,000	
1p13.2	Loss	115,300,000	116,000,000	
2p23.3	Gain	24,000,000	24,300,000	
2p23.3	Loss	27,100,000	27,300,000	
2p23.3	Loss	27,400,000	27,600,000	
3q12.2 to q13.31	Complex gains and losses consisten with chromothripsis	100,700,000	116,800,000	
3q13.32 to q21.3	Complex gains and losses consisten with chromothripsis	118,300,000	127,600,000	
3q22.1	Gain	129,700,000	129,900,000	
3q27.2	Gain	185,400,000	185,600,000	
3q29 to qter	Loss	196,900,000	197,800,000	Terminal
5pter to p13.3	Loss	1	32,000,000	Terminal
5p13.2	Loss	38,000,000	39,700,000	
5q23.1	Gain	119,900,000	120,300,000	
5q23.1	Gain	121,000,000	121,500,000	
5q31.1	Loss	130,800,000	131,900,000	
5q31.2	Loss	136,900,000	138,600,000	
6p12.3 to q13	Homozygous	49,100,000	71,400,000	
6q25.3	Loss	156,400,000	156,900,000	
6q25.3	Loss	157,500,000	158,000,000	
7p12.2 to q11.22	Complex gains and losses consisten with chromothripsis	61,900,000	70,000,000	
7q21.11 to q26.3	Loss	86,200,000	159,100,000	
16q11.2 to q24.1	Loss	46,500,000	86,300,000	
16q24.3 to qter	Loss	89,500,000	90,300,000	Terminal
17pter to p11.2	Loss	1	19,100,000	Terminal
17q11.1 to q12	Abnormal (low level mosaic)	25,160,000	37,500,000	Very low level mosaicism- only slightly w
20q11.23 to q13.13	Loss	35,300,000	49,200,000	
XY characteristic of Male Genotype				
*Majority of Losses seem to be at a frequency of ~75-85%; Frequency of gains are not able to be accurately approximated visually				

Chromoanagenesis (3q12.2 to q13.31; 3q13.32 to q21.3)

Chromoanagenesis (7p12.2 to q11.22)

Chr.	Approximate Bkpts	Abnormality	Start	Stop	Size
3	3q12.2 to q13.31	Loss	100,712,059	104,778,910	4,066,851
3	chromothripsis	Gain*	104,890,715	105,476,196	585,481
3		Gain*	113,370,010	113,495,648	125,638
3		Gain*	115,543,891	115,704,434	160,543
3		Loss	115,721,099	116,712,193	991,094

3	3q13.32 to q21.3	Gain? - Very low freq	118,281,297	118,530,692	249,395
3	chromothripsis	Gain*	119,433,300	121,931,055	2,517,755
3		Gain*	122,648,823	125,683,874	3,088,097
		Gain* (small in size)	125,844,981	125,899,367	54,386
3		Gain (small in size)	126,252,506	126,257,356	4,850
3		Gain	126,440,248	127,475,626	1,045,378

7	7q11.22	Gain*	62,012,221	62,577,770	592,187
7	chromothripsis	Gain*	63,431,014	68,270,995	4,839,981
7		Gain*	68,504,050	69,244,026	739,976
7		Gain	69,437,047	69,472,821	35,774
7		Loss	69,475,046	69,649,683	174,637
7		Loss	71,000,614	71,918,506	917,892

The SNP microarray data was based on the hg19 (GRCh37) genome assembly.

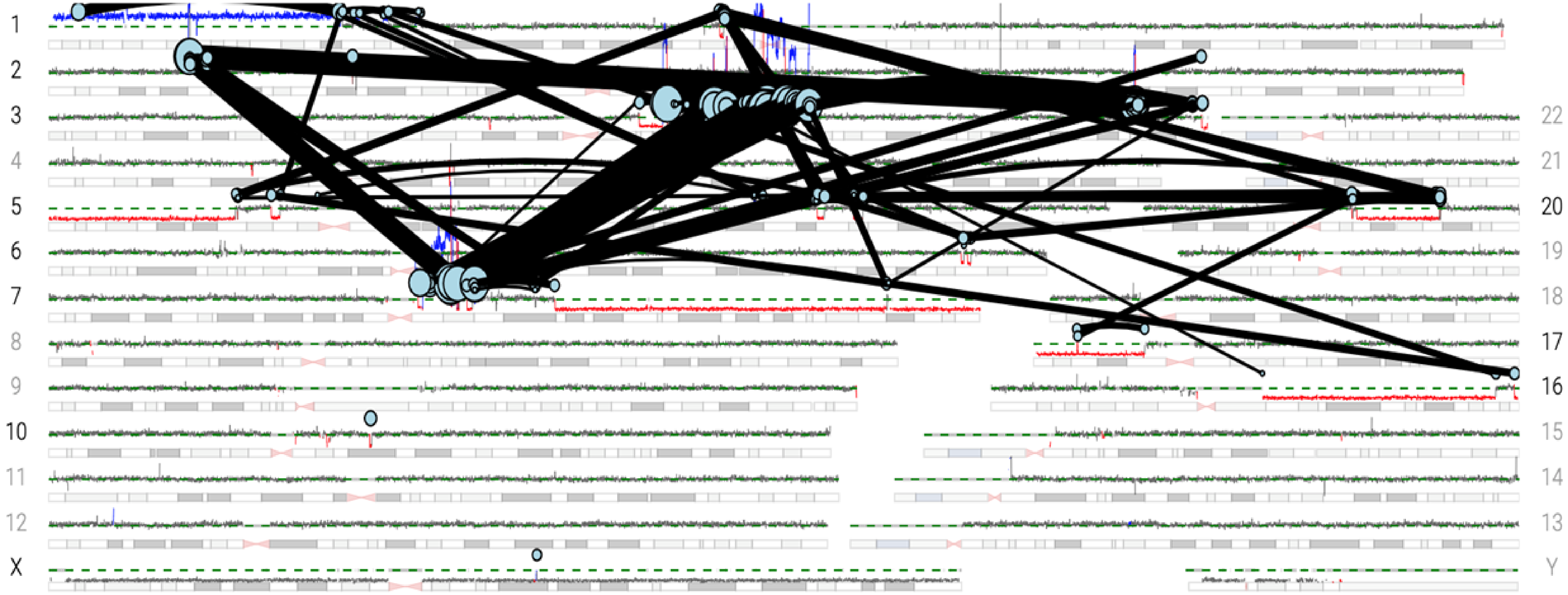

Supplementary Figure S1: Genome-wide plot of mate pair sequencing. Black lines show rearrangements, blue lines are gains/amplifications, and red lines are losses.

Supplementary Table S3: Mate pair sequencing data involving chromosomes 2, 3, and 7.

ID	ChrA	ChrB	PosA	PosB	LocusA	LocusB	GeneA	GeneA Strand	GeneB	GeneB strand	Gene prediction
1	2	2	24176453	27091102	2p23.3	2p23.3	FAM228A	+	KHK	+	
2	2	2	26599130	27332156	2p23.3	2p23.3	CIB4	-	GTF3C2	-	fusion GTF3C2->CIB4
3	2	2	51937034	52000037	2p16.3	2p16.3	LOC730100	+	LOC730100	+	
4	2	3	24108735	185641841	2p23.3	3q27.2	FAM228B	+	IGF2BP2	-	
5	2	3	26824472	184932817	2p23.3	3q27.2	no_gene	NA	VPS8	+	truncation noGene->VPS8
6	2	3	24124069	195494223	2p23.3	3q29	FAM228B	+	no_gene	NA	truncation FAM228B->noGene
7	2	3	24162985	195523738	2p23.3	3q29	FAM228B	+	PPP1R2	-	
8	2	3	27143574	197217638	2p23.3	3q29	no_gene	NA	DLG1	-	truncation DLG1->noGene
9	2	7	23842879	69782018	2p23.3	7q11.22	ATAD2B	-	AUTS2	+	
10	2	7	24175563	70186455	2p23.3	7q11.22	FAM228A	+	AUTS2	+	fusion FAM228A->AUTS2
11	2	7	26600038	71276606	2p23.3	7q11.22	CIB4	-	GALNT17	+	fusion CIB4->GALNT17
12	2	7	23843471	83249803	2p23.3	7q21.11	ATAD2B	-	no_gene	NA	truncation noGene->ATAD2B
13	2	7	24124701	83226322	2p23.3	7q21.11	FAM228B	+	no_gene	NA	truncation noGene->FAM228B
14	2	7	26893424	83264026	2p23.3	7q21.11	DPYSL5	+	no_gene	NA	truncation DPYSL5->noGene
15	2	7	197253508	66603932	2q33.1	7q11.21	ANKRD44	-	no_gene	NA	truncation noGene->ANKRD44
16	3	3	100992219	116994946	3q12.2	3q13.31	ABI3BP	-	no_gene	NA	truncation noGene->ABI3BP
17	3	3	105065526	115999831	3q13.11	3q13.31	no_gene	NA	LSAMP	-	truncation noGene->LSAMP
18	3	3	105767086	122796155	3q13.11	3q21.1	CBLB	-	SLC49A4	+	
19	3	3	115830085	120679688	3q13.31	3q13.33	LSAMP	-	HGD	-	fusion HGD->LSAMP
20	3	3	113789209	122823427	3q13.31	3q21.1	ATP6V1A	+	SLC49A4	+	
21	3	3	113776640	126531069	3q13.31	3q21.3	ATP6V1A	+	C3orf22	-	fusion C3orf22->ATP6V1A
22	3	3	113640674	126710079	3q13.2	3q21.3	no_gene	NA	CHCHD6	+	truncation noGene->CHCHD6
23	3	3	115819999	125950859	3q13.31	3q21.2	LSAMP	-	no_gene	NA	truncation LSAMP->noGene
24	3	3	120838373	121057786	3q13.33	3q13.33	LINC02049	-	STXBP5L	+	
25	3	3	115820115	126709599	3q13.31	3q21.3	LSAMP	-	CHCHD6	+	
26	3	3	119714040	122933937	3q13.33	3q21.1	MAATS1	+	SEMA5B	-	fusion SEMA5B->MAATS1
27	3	3	120668518	122055860	3q13.33	3q13.33	HGD	-	CD86	+	
28	3	3	122233504	125950401	3q21.1	3q21.2	CASR	+	no_gene	NA	truncation CASR->noGene
29	3	3	121735209	126937593	3q13.33	3q21.3	GOLGB1	-	CHCHD6	+	fusion GOLGB1->CHCHD6
30	3	3	125975069	126144322	3q21.2	3q21.3	ROPN1B	+	ALDH1L1	-	fusion ROPN1B->ALDH1L1
31	3	3	125950869	126707958	3q21.2	3q21.3	no_gene	NA	CHCHD6	+	truncation noGene->CHCHD6
32	3	3	127759498	130110454	3q21.3	3q22.1	MGLL	-	FAM86HP	-	
33	3	3	127841422	185641682	3q21.3	3q27.2	no_gene	NA	IGF2BP2	-	truncation noGene->IGF2BP2
34	3	3	127757496	185894322	3q21.3	3q27.2	MGLL	-	no_gene	NA	truncation MGLL->noGene
35	3	3	127966149	186702647	3q21.3	3q27.3	KBTBD12	+	no_gene	NA	truncation KBTBD12->noGene
36	3	3	130110492	185894391	3q22.1	3q27.2	FAM86HP	-	no_gene	NA	truncation noGene->FAM86HP
37	3	3	129923029	197196935	3q22.1	3q29	no_gene	NA	DLG1	-	truncation noGene->DLG1
38	3	3	186247721	186703274	3q27.2	3q27.3	DGKG	-	no_gene	NA	truncation noGene->DGKG
39	3	3	186248185	195553109	3q27.2	3q29	DGKG	-	no_gene	NA	truncation DGKG->noGene
40	3	3	195524772	197217356	3q29	3q29	PPP1R2	-	DLG1	-	fusion PPP1R2->DLG1
41	3	7	113770973	68819537	3q13.31	7q11.22	ATP6V1A	+	no_gene	NA	truncation ATP6V1A->noGene
42	3	7	113788574	68806912	3q13.31	7q11.22	ATP6V1A	+	no_gene	NA	truncation ATP6V1A->noGene
43	3	7	120840939	64772810	3q13.33	7q11.21	no_gene	NA	no_gene	NA	
44	3	7	121057437	65186429	3q13.33	7q11.21	STXBP5L	+	INTS4P1	+	fusion STXBP5L->INTS4P1
45	3	7	121185065	65214917	3q13.33	7q11.21	STXBP5L	+	INTS4P1	+	fusion STXBP5L->INTS4P1
46	3	7	118816864	67734630	3q13.32	7q11.22	no_gene	NA	no_gene	NA	
47	3	7	122105372	65022821	3q13.33	7q11.21	CD86	+	no_gene	NA	truncation CD86->noGene
48	3	7	122056438	66695938	3q13.33	7q11.21	CD86	+	RABGEF1	+	fusion RABGEF1->CD86
49	3	7	124384374	65212653	3q21.2	7q11.21	KALRN	+	INTS4P1	+	
50	3	7	121045503	68782233	3q13.33	7q11.22	STXBP5L	+	no_gene	NA	truncation noGene->STXBP5L
51	3	7	122822764	68806396	3q21.1	7q11.22	SLC49A4	+	no_gene	NA	truncation SLC49A4->noGene
52	3	7	122873319	68810842	3q21.1	7q11.22	SLC49A4	+	no_gene	NA	truncation noGene->SLC49A4
53	3	7	126920746	65184976	3q21.3	7q11.21	CHCHD6	+	INTS4P1	+	fusion INTS4P1->CHCHD6
54	3	7	126125574	68722206	3q21.3	7q11.22	ALDH1L1	-	no_gene	NA	truncation ALDH1L1->noGene
55	3	7	126543137	68806186	3q21.3	7q11.22	C3orf22	-	no_gene	NA	truncation noGene->C3orf22
56	3	7	127883676	71530453	3q21.3	7q11.22	no_gene	NA	GALNT17	+	truncation GALNT17->noGene
57	3	7	129924506	70009817	3q22.1	7q11.22	no_gene	NA	AUTS2	+	truncation AUTS2->noGene
58	3	7	127757516	72900295	3q21.3	7q11.23	MGLL	-	POM121	+	fusion POM121->MGLL
59	3	7	127841040	72915403	3q21.3	7q11.23	no_gene	NA	POM121	+	truncation POM121->noGene
60	3	7	127883214	83235491	3q21.3	7q21.11	no_gene	NA	no_gene	NA	
61	3	7	184932250	71276233	3q27.2	7q11.22	VPS8	+	GALNT17	+	
62	3	7	197016845	72915877	3q29	7q11.23	MELTF	-	POM121	+	fusion MELTF->POM121
63	3	7	197245041	72827007	3q29	7q11.23	DLG1	-	TYW1B	-	
64	3	7	130118482	143404806	3q22.1	7q35	LINC02021	+	EPHA1	-	fusion EPHA1->LINC02021
65	7	7	63118805	63955133	7q11.21	7q11.21	no_gene	NA	no_gene	NA	
66	7	7	68721805	69036211	7q11.22	7q11.22	no_gene	NA	no_gene	NA	
67	7	7	69970218	72885224	7q11.22	7q11.23	AUTS2	+	POM121	+	
68	7	7	72468855	72956717	7q11.22	7q11.23	no_gene	NA	NSUN5P2	-	truncation NSUN5P2->noGene
69	7	7	71535763	86586652	7q11.22	7q21.11	GALNT17	+	no_gene	NA	truncation noGene->GALNT17

Chr = chromosome; Pos = position; The genomic locations are based on the GRCh38 genome assembly.

Supplementary Table S4: Structural variants involving chromosomes 2, 3, and 7 by nanopore sequencing.

ID	Breakpoints of structrual variants						crRNA	Copy number variants	Exon, intron, intergenic regions
	Chr.	Genomic location							
1	3	chr3:100,599,929	chr3:100,626,019				1	Gain	Intron (*ABI3BP*)
2	3	chr3:104,858,021	chr3:104,892,347	chr3:104,908,408			2	Gain, amplification	Intergenic
3*	3	chr3:105,412,208	chr3:105,474,400	chr3:105,486,522*			3	Gain, amplification	Intron (*CBLB*)
4	3	chr3:105,966,313	chr3:105,966,666					Loss	Intergenic
5*	3	chr3:113,359,485*	chr3:113,371,858	chr3:113,374,867			4	Loss, gain, gain	Intergenic, exon (*USF3*), exon (*USF3*)
6*	3	chr3:113,494,388*	chr3:113,508,181*				5	Gain, loss	Intron (*ATP6V1A*)
7	3	chr3:115,167,760	chr3:115,167,828					Loss	Intergenic
8*	3	chr3:115,538,739*	chr3:115,544,900*				6	Loss, gain, amplification	Intron (*LSAMP*)
9*	3	chr3:115,674,519	chr3:115,702,634	chr3:115,718,859*			7	Gain, gain, loss	Intron (*LSAMP*)
10	3	chr3:116,746,814	chr3:116,769,363					Normal	Intergenic
11	3	chr3:116,821,197	chr3:116,841,964	chr3:116,849,099			8	Normal	Intergenic
12	3	chr3:118,252,153	chr3:118,281,575	chr3:118,281,817	chr3:118,282,696	chr3:118,291,381	9	Normal	Intron (*NR_135547.1*)
13*	3	chr3:119,401,320	chr3:119,432,884*	chr3:119,434,664			11	Normal, gain	Intergenic, intron (*CFAP91*), intron (*CFAP91*)
14*	3	chr3:121,890,413	chr3:121,927,745	chr3:121,952,434*			12	Gain, amplification, gain, loss	Intergenic, intron, (*CASR*), intron (*CASR*)
15*	3	chr3:122,515,022*	chr3:122,542,323*					Amplification, loss	Intron (*SLC49A4*)
16*	3	chr3:122,592,305*	chr3:122,611,656	chr3:122,640,962	chr3:122,652,788*	chr3:122,681,826	13	Normal, gain, normal, gain, normal	Intron (*SLC49A4*), intergenic, exon (*SEMA5B*), intron (*SEMA5B*), intron (*SEMA5B*)
17	3	chr3:123,664,618	chr3:123,716,995					Gain	Intron (*CCDC14*), intergenic
18*	3	chr3:125,624,678	chr3:125,669,270*	chr3:125,669,766*	chr3:125,672,194			Gain, amplification	Intergenic, intron (*ALG1L*), intron (*ALG1L*), intron (*ALG1L*)
19*	3	chr3:125,675,646	chr3:125,682,047	chr3:125,694,156*			14	Gain, amplification, gain, amplification, normal	Intron (*ALG1L*)
20*	3	chr3:125,844,441*	chr3:125,846,229	chr3:125,863,212*			15	Gain, amplification	Intron (*ALD1H1*)
21	3	chr3:125,897,574	chr3:125,922,069				16	Normal	Intron (*ALD1H1*)
22*	3	chr3:126,249,843*	chr3:126,255,019				17	Normal	Intron (*CHST13*)
23*	3	chr3:126,256,677	chr3:126,258,740	chr3:126,262,047*			18	Normal	Intron (*CHST13*), intron (*CHST13*), exon (*CHST13*)
24*	3	chr3:126,424,358	chr3:126,424,702	chr3:126,426,808*	chr3:126,428,508*			Normal	Intron (*CHCHD6*)
25*	3	chr3:126,428,828*	chr3:126,441,362				19	Gain	Intron (*CHCHD6*)
26*	3	chr3:127,383,619	chr3:127,407,727	chr3:127,476,304*			20	Normal, gain, normal	Intron (*PODXL2*), intron (*MGLL*), intron (*MGLL*)
27*	3	chr3:127,602,417*	chr3:127,639,712					Normal	Intergenic, intron (*KBTBD12*)
28*	3	chr3:129,643,392*	chr3:129,690,495					Gain	Intergenic
29	7	chr7:317,478	chr7:336,683	chr7:337,238	chr7:342,677			Normal	Intergenic
30	7	chr7:61,968,673	chr7:61,969,730	chr7:62,020,349			21	Normal, normal, gain, amplification	Intergenic
31*	7	chr7:63,415,194*	chr7:63,435,841	chr7:63,451,382			23	Loss, gain, gain	Intergenic
32*	7	chr7:68,160,491	chr7:68,187,000*	chr7:68,187,235*	chr7:68,226,475	chr7:68,268,667	24	normal, gain, loss, gain, normal, amplification, loss, gain	Intergenic
		chr7:68,271,287*	chr7:68,271,421*	chr7:68,271,991*					
33*	7	chr7:68,500,503*	chr7:68,505,483				25	Gain	Intergenic
34*	7	chr7:69,202,729	chr7:69,242,939	chr7:69,246,688*			26	Gain, normal	Intron (*AUTS2*)
35*	7	chr7:69,435,177*	chr7:69,438,275	chr7:69,468,534	chr7:69,469,200		27	Gain, normal, gain	Intron (*AUTS2*)
36*	7	chr7:69,471,074	chr7:69,474,861*				28/29	Gain, loss	Intron (*AUTS2*)
37*	7	chr7:70,992,992	chr7:70,995,713*	chr7:71,005,705	chr7:71,006,139		30	Normal, loss, loss, loss	Intron (*GALNT17*)
38*	7	chr7:72,355,740*	chr7:72,370,519*					Normal	Intron (*POM121*)
39*	2	chr2:24,065,657*	chr2:24,069,092	chr2:24,069,719	chr2:24,093,039			Gain, normal, amplification	Intron (*ATAD2B*)
40	2	chr2:109,815,783	chr2:109,816,313					Gain	Intron (*SH3RF3*)
41	2	chr2:190,907,264	chr2:190,932,292	chr2:190,946,998				Normal	Intergenic

* breakpoint detected by both Mate Pair sequencing and nanopore sequencing

The Genomic locations are based on the hg19 (GRCh37) genome assembly.

Supplementary Table S5: DNA sequences flanking breakpoints of structural variants.

ID	Position	Genomic Location	Strand (+/-)	Intron / Exon	Name	Family	Class	Repeatmasker
1	chr3:122652784	*SEMA5B*	-	intron	AluSx	Alu	SINE	HAL1
2	chr3:119432887	*CFAP91*	+	intron	L1ME4a	L1	LINE	L1ME4a
3	chr2:24066341	*ATAD2B*	-	intron	L1MEa	L1	LINE	L1MEa
4	chr2:24065749	*ATAD2B*	-	intron	L1MEa	L1	LINE	L1MEa
5	chr7:72370832	*POM121*	+	intron	L1MB4	L1	LINE	L1MB4
6	chr7:72355763	*POM121*	+	intron	L1MEc	L1	LINE	L1MEc
7	chr3:125693912	*ROPN1B*	+	intron	FRAM	Alu	SINE	N/A
8	chr3:105485930	*CBLB*	-	intron	N/A	N/A	N/A	N/A
9	chr3:121952351	*CASR*	+	intron	N/A	N/A	N/A	N/A
10	chr3:115718678	*LSAMP*	-	intron	N/A	N/A	N/A	N/A
11	chr3:115548932	*LSAMP*	-	intron	N/A	N/A	N/A	N/A
12	chr3:115538846	*LSAMP*	-	intron	N/A	N/A	N/A	N/A
13	chr3:115538962	*LSAMP*	-	intron	N/A	N/A	N/A	N/A
14	chr3:113508056	*ATP6V1A*	+	intron	AluSq	Alu	SINE	N/A
15	chr3:113495487	*ATP6V1A*	+	intron	N/A	N/A	N/A	N/A
16	chr3:113507421	*ATP6V1A*	+	intron	AluSq2	Alu	SINE	N/A
17	chr3:113489820	*ATP6V1A*	+	intron	AluSx3	Alu	SINE	N/A
18	chr3:127476339	*MGLL*	-	intron	N/A	N/A	N/A	N/A
19	chr3:127478341	*MGLL*	-	intron	N/A	N/A	N/A	N/A
20	chr3:127476359	*MGLL*	-	intron	N/A	N/A	N/A	N/A
21	chr7:69474803	*AUTS2*	+	intron	MIRc	MIR	SINE	N/A
22	chr7:69435204	*AUTS2*	+	intron	N/A	N/A	N/A	N/A
23	chr7:69247004	*AUTS2*	+	intron	MER5A	hAT-Charlie	DNA	N/A
24	chr3:125863165	*ALDH1L1*	-	intron	L1MB1	L1	LINE	L1MB1
25	chr3:125844417	*ALDH1L1*	-	intron	N/A	N/A	N/A	N/A
26	chr3:126428922	*CHCHD6*	+	intron	MER58A	hAT-Charlie	DNA	L1MB7
27	chr3:126428442	*CHCHD6*	+	intron	L1MB7	L1	LINE	L1MB7
28	chr3:126426801	*CHCHD6*	+	intron	L2b	L2	LINE	N/A
29	chr3:122541611	*SLC49A4*	+	intron	L2a	L2	LINE	L2a
30	chr3:122592166	*SLC49A4*	+	intron	AluSz	Alu	SINE	N/A
31	chr3:122515002	*SLC49A4*	+	intron	N/A	N/A	N/A	N/A
32	chr3:122542274	*SLC49A4*	+	intron	N/A	N/A	N/A	N/A
33	chr7:70995438	*GALNT17*	+	intron	MER5A	hAT-Charlie	DNA	MER5A
34	chr7:71000748	*GALNT17*	+	intron	N/A	N/A	N/A	N/A
35	chr3:126249912	*CHST13*	+	intron	N/A	N/A	N/A	N/A
36	chr3:126261980	*CHST13*	+	exon	N/A	N/A	N/A	N/A
37	chr7:68284524	Intergenic region	N/A	N/A	L3	CR1	LINE	L3
38	chr3:113359521	Intergenic region	N/A	N/A	Charlie1a	hAT-Charlie	DNA	L1MEg, Charlie1a
39	chr3:104784370	Intergenic region	N/A	N/A	L1ME1	L1	LINE	L1ME1
40	chr3:127602519	Intergenic region	N/A	N/A	AluSz	Alu	SINE	N/A
41	chr3:127602057	Intergenic region	N/A	N/A	N/A	N/A	N/A	N/A
42	chr7:68271899	Intergenic region	N/A	N/A	AluSx1	Alu	SINE	N/A
43	chr7:68247220	Intergenic region	N/A	N/A	AluSp	Alu	SINE	N/A
44	chr7:68271383	Intergenic region	N/A	N/A	AluSz6	Alu	SINE	N/A
45	chr7:68275829	Intergenic region	N/A	N/A	AluSx	Alu	SINE	N/A
46	chr7:68187193	Intergenic region	N/A	N/A	N/A	N/A	N/A	N/A
47	chr7:68271173	Intergenic region	N/A	N/A	L3	CR1	LINE	N/A
48	chr7:68501198	Intergenic region	N/A	N/A	N/A	N/A	N/A	N/A
49	chr7:68186792	Intergenic region	N/A	N/A	AluSg7	Alu	SINE	N/A
50	chr3:125669712	Intergenic region	N/A	N/A	N/A	N/A	N/A	N/A
51	chr3:129641872	Intergenic region	N/A	N/A	AluSx	Alu	SINE	N/A
52	chr3:125669702	Intergenic region	N/A	N/A	N/A	N/A	N/A	N/A
53	chr3:125669244	Intergenic region	N/A	N/A	MLTD1	ERVL-MaLR	LTR	N/A
54	chr3:129643349	Intergenic region	N/A	N/A	AluSz	Alu	SINE	N/A
55	chr7:63415511	Intergenic region	N/A	N/A	N/A	N/A	N/A	N/A

N/A: not available. The genomic locations are based on the GRCh38 genome assembly.
